# Supplementary material for: Destabilizing effects on a classic tri-trophic oyster-reef cascade
Source: PLoS One. 2020 Dec 15;15(12):e0242965. doi: 10.1371/journal.pone.0242965 (PMC7737904; doi:10.1371/journal.pone.0242965)
Supplement: S1 Table — Paired t-tests between numbers of small vs. large oysters consumed across treatments for the MPE experiment. N = 10 for every treatment; N = 5 each representing each substrate type within each treatment. Rejection P = the Holm-Bonferroni sequential adjusted value for that comparison. No test is significant at familywise 0.05 level based on adjusted P. Mean differences (i.e., absolute value) range from 0.4 to 1.9 out of a maximum of 15 possible (i.e., 15 small and 15 large) across treatments. (DOCX) [file pone.0242965.s001.docx]

**S1 Table. Small vs. large oysters consumed across MPE treatments.** Paired t-tests between numbers of small vs. large oysters consumed across treatments for the MPE experiment. N = 10 for every treatment; N = 5 each representing each substrate type within each treatment. Rejection P = the Holm-Bonferroni sequential adjusted value for that comparison. No test is significant at familywise 0.05 level based on adjusted P. Mean differences (i.e., absolute value) range from 0.4 to 1.9 out of a maximum of 15 possible (i.e., 15 small and 15 large) across treatments.

| **Treatment** | **Num Small** | **Num Large** | **Mean Diff** | **Paired-t** | **P** | **Rejection P** |
| --- | --- | --- | --- | --- | --- | --- |
| SC | 5.5 | 6.7 | -1.200 | -2.449 | 0.0370 | 0.0083 |
| MC | 11.2 | 9.3 | 1.900 | 2.310 | 0.0460 | 0.0100 |
| TF/MC | 2.2 | 1.5 | 0.700 | 1.909 | 0.0890 | 0.0125 |
| TF/SC | 6.2 | 7.9 | -1.700 | -1.645 | 0.1340 | 0.0167 |
| SC/MC | 7.6 | 6.5 | 1.100 | 1.222 | 0.2530 | 0.0250 |
| TF/SC/MC | 8.3 | 7.9 | 0.400 | 0.349 | 0.7350 | 0.0500 |
