# Supplementary material for: Destabilizing effects on a classic tri-trophic oyster-reef cascade
Source: PLoS One. 2020 Dec 15;15(12):e0242965. doi: 10.1371/journal.pone.0242965 (PMC7737904; doi:10.1371/journal.pone.0242965)

PLOS ONE

Destabilizing effects on a classic tri-trophic oyster-reef cascade

--Manuscript Draft--

**Manuscript Number:**	PONE-D-20-05873
**Article Type:**	Research Article
**Full Title:**	Destabilizing effects on a classic tri-trophic oyster-reef cascade
**Short Title:**	Destabilizing effects on a classic trophic cascade
**Corresponding Author:**	Chet F. Rakocinski, Ph.D.<br>University of Southern Mississippi<br>Ocean Springs, MS UNITED STATES
**Keywords:**	Oyster reefs; trophic cascades; multiple predator effects; Trait-Mediated Indirect Interactions, non-consumptive effects
**Abstract:**	How multiple predators interact to stabilize or destabilize trophic cascades is currently of intensive ecological interest. To examine this question relative to the classic tri-trophic oyster reef cascade, we manipulated configurations of three predators, including Gulf toadfish, Gulf stone crab, and mud crab, as well as limestone and oyster-shell substrates. In addition, we performed follow-up trait-mediated-indirect interaction (TMII) experiments to compare behavioral non-consumptive effects (NCEs) on oyster consumption with respect to mud-crab size and type of top predator. We found that the classic tri-trophic cascade formed by the toadfish-mud crab-oyster association was potentially weakened by several interactions. Consumption of oysters and mud crabs by the generalist stone crab was undeterred by toadfish. Although feeding by mud crabs was suppressed in the presence of either toadfish or stone crabs, non-consumptive effects (NCEs) were much weaker for stone crabs. Consequently, the sum total effect was destabilizing when all three predator species were together. Weaker avoidance responses by mud crabs to the presence of stone crabs culminated in relatively high direct predation on mud crabs. TMII experiments confirmed weaker avoidance of mud crab to stone crabs than to toadfish, and revealed that NCEs with respect to oyster consumption by mud crabs in the MPE experiment were mostly due to behavioral suppression, rather than to interference. An inverse relationship between mud crab size and NCE strength potentially contributes an additional destabilizing influence on the tri-trophic-cascade. Finally, although greater habitat complexity generally dampened the consumption of oysters across treatments, complex habitat potentially destabilized the tri-trophic cascade by relaxing suppression of feeding by mud crabs. This study underscores how food web stability is mediated by ecological interactions that can weaken or reinforce trophic cascades under variable natural conditions.
**Order of Authors:**	Virginia R. Fleer
	Chet F. Rakocinski, Ph.D.
**Additional Information:**	
**Question**	**Response**
**Financial Disclosure**<br><br>Enter a financial disclosure statement that describes the sources of funding for the work included in this submission. Review the submission guidelines for detailed requirements. View published research articles from *PLOS ONE* for specific examples.<br><br>This statement is required for submission	Research for this article was conducted as part of a Ph.D. Dissertation by VRF, and was not supported by any specific grant from funding agencies in the public, commercial, or not-for-profit sectors. Supplies for this research project were largely provided to VRF by the Mississippi Department of Marine Resources.

Powered by Editorial Manager® and ProduXion Manager® from Aries Systems Corporation

and **will appear in the published article** if the submission is accepted. Please make sure it is accurate.<br><br>**Unfunded studies**<br>Enter: *The author(s) received no specific funding for this work.*<br><br>**Funded studies**<br>Enter a statement with the following details:<br>• Initials of the authors who received each award<br>• Grant numbers awarded to each author<br>• The full name of each funder<br>• URL of each funder website<br>• Did the sponsors or funders play any role in the study design, data collection and analysis, decision to publish, or preparation of the manuscript?<br>• **NO** - Include this sentence at the end of your statement: *The funders had no role in study design, data collection and analysis, decision to publish, or preparation of the manuscript.*<br>• **YES** - Specify the role(s) played.<br><br>* typeset	
**Competing Interests**<br><br>Use the instructions below to enter a competing interest statement for this submission. On behalf of all authors, disclose any competing interests that could be perceived to bias this work—acknowledging all financial support and any other relevant financial or non-financial competing interests.<br><br>This statement **will appear in the published article** if the submission is accepted. Please make sure it is accurate. View published research articles from *PLOS ONE* for specific examples.	The authors have no competing interests.

Powered by Editorial Manager® and ProduXion Manager® from Aries Systems Corporation

**NO authors have competing interests**<br><br>Enter: *The authors have declared that no competing interests exist.*<br><br>**Authors with competing interests**<br><br>Enter competing interest details beginning with this statement:<br><br>*I have read the journal's policy and the authors of this manuscript have the following competing interests: [insert competing interests here]*<br><br>* typeset	
**Ethics Statement**<br><br>Enter an ethics statement for this submission. This statement is required if the study involved:<br><br>• Human participants<br>• Human specimens or tissue<br>• Vertebrate animals or cephalopods<br>• Vertebrate embryos or tissues<br>• Field research<br><br>Write "N/A" if the submission does not require an ethics statement.<br><br>General guidance is provided below. Consult the submission guidelines for detailed instructions. **Make sure that all information entered here is included in the Methods section of the manuscript.**	This study was carried out in strict accordance with the recommendations in the Guide for the Care and Use of Laboratory Animals of the National Institutes of Health. The protocol for this study was approved by the Institutional Animal Care and Use Committee (IUCUC) of the University of Southern Mississippi (Protocol Number: 16052606), based on an Animal Subjects Research Application submitted to the USM Office of Research Integrity. In addition, VRF completed the Collaborative Institutional Training Initiative Program (CITI Program). Certificates were obtained for both the animal subjects course and the common course covering various considerations related to ethical conduct in all professional and research activities. All efforts to obtain, handle and house fish and invertebrate subjects were made humanely. Following the completion of experiments, fish subjects were released back into the wild. Field work was not done in protected or privately owned areas, and no protected species were sampled. Collections of reef animals were made under the auspices of a Scientific Research Permit issued to the USM Gulf Coast Research Laboratory by the Mississippi Department of Marine Resources (MSDMR).

Powered by Editorial Manager® and ProduXion Manager® from Aries Systems Corporation

**Format for specific study types**<br><br>**Human Subject Research (involving human participants and/or tissue)**<br>• Give the name of the institutional review board or ethics committee that approved the study<br>• Include the approval number and/or a statement indicating approval of this research<br>• Indicate the form of consent obtained (written/oral) or the reason that consent was not obtained (e.g. the data were analyzed anonymously)<br><br>**Animal Research (involving vertebrate animals, embryos or tissues)**<br>• Provide the name of the Institutional Animal Care and Use Committee (IACUC) or other relevant ethics board that reviewed the study protocol, and indicate whether they approved this research or granted a formal waiver of ethical approval<br>• Include an approval number if one was obtained<br>• If the study involved *non-human primates*, add *additional details* about animal welfare and steps taken to ameliorate suffering<br>• If anesthesia, euthanasia, or any kind of animal sacrifice is part of the study, include briefly which substances and/or methods were applied<br><br>**Field Research**<br><br>Include the following details if this study involves the collection of plant, animal, or other materials from a natural setting:<br>• Field permit number<br>• Name of the institution or relevant body that granted permission	
**Data Availability**<br><br>Authors are required to make all data underlying the findings described fully available, without restriction, and from the time of publication. PLOS allows rare exceptions to address legal and ethical concerns. See the PLOS Data Policy and FAQ for detailed information.	Yes - all data are fully available without restriction

<table>
<tr><td>A Data Availability Statement describing where the data can be found is required at submission. Your answers to this question constitute the Data Availability Statement and will be published in the article, if accepted.

Important: Stating ‘data available on request from the author’ is not sufficient. If your data are only available upon request, select ‘No’ for the first question and explain your exceptional situation in the text box.

Do the authors confirm that all data underlying the findings described in their manuscript are fully available without restriction?</td><td></td></tr>
<tr><td>Describe where the data may be found in full sentences. If you are copying our sample text, replace any instances of XXX with the appropriate details.

• If the data are held or will be held in a public repository, include URLs, accession numbers or DOIs. If this information will only be available after acceptance, indicate this by ticking the box below. For example: All XXX files are available from the XXX database (accession number(s) XXX, XXX.).
• If the data are all contained within the manuscript and/or Supporting Information files, enter the following: All relevant data are within the manuscript and its Supporting Information files.
• If neither of these applies but you are able to provide details of access elsewhere, with or without limitations, please do so. For example:

Data cannot be shared publicly because of [XXX]. Data are available from the XXX Institutional Data Access / Ethics Committee (contact via XXX) for researchers who meet the criteria for access to confidential data.

The data underlying the results presented in the study are available from (include the name of the third party</td><td>Data used for this study were obtained from experiments as described in the Methods and materials section of this article. All data were collected, compiled, curated and validated by V.R. Fleer. If accepted for publication, data used for this study, including metadata formats, will be submitted as excel files exported from SPSS 25.0 files to the GRIIDC data archive, at https://data.gulfresearchinitiative.org/, hosted by the Harte Research Institute for Gulf of Mexico Studies of Texas A&M University-Corpus Christi and The Gulf of Mexico Research Initiative (GoMRI).</td></tr>
</table>

Powered by Editorial Manager® and ProduXion Manager® from Aries Systems Corporation

and contact information or URL).<br>• This text is appropriate if the data are owned by a third party and authors do not have permission to share the data.<br><br>* typeset	
Additional data availability information:	Tick here if the URLs/accession numbers/DOIs will be available only after acceptance of the manuscript for publication so that we can ensure their inclusion before publication.

Manuscript

Click here to access/download;Manuscript;PLoSOne_Destabilizing effects on

Destabilizing effects on a classic tri-trophic oyster-reef cascade

Virginia R. Fleer, Chet F. Rakocinski*

Division of Coastal Sciences, School of Ocean Science and Engineering, University of Southern Mississippi, Gulf Coast Research Laboratory, 703 East Beach Drive, Ocean Springs, MS 39564, United States of America

*Corresponding author: Tel.: 1-228-872-4284, Email: chet.rakocinski@usm.edu

Abstract

How multiple predators interact to stabilize or destabilize trophic cascades is currently of intensive ecological interest. To examine this question relative to the classic tri-trophic oyster reef cascade, we manipulated configurations of three predators, including Gulf toadfish, Gulf stone crab, and mud crab, as well as limestone and oyster-shell substrates. In addition, we performed follow-up trait-mediated-indirect interaction (TMII) experiments to compare behavioral non-consumptive effects (NCEs) on oyster consumption with respect to mud-crab size and type of top predator. We found that the classic tri-trophic cascade formed by the toadfish-mud crab-oyster association was potentially weakened by several interactions. Consumption of oysters and mud crabs by the generalist stone crab was undeterred by toadfish. Although feeding by mud crabs was suppressed in the presence of either toadfish or stone crabs, non-consumptive effects (NCEs) were much weaker for stone crabs. Consequently, the sum total effect was destabilizing when all three predator species were together. Weaker avoidance responses by mud crabs to the presence of stone crabs culminated in relatively high direct predation on mud crabs. TMII experiments confirmed weaker avoidance of mud crab to stone crabs than to toadfish, and revealed that NCEs with respect to oyster consumption by mud crabs in the MPE experiment were mostly due to behavioral suppression, rather than to interference. An inverse relationship between mud crab size and NCE strength potentially contributes an additional destabilizing influence on the tri-trophic-cascade. Finally, although greater habitat complexity generally dampened the consumption of oysters across treatments, complex habitat potentially destabilized the tri-trophic cascade by relaxing suppression of

feeding by mud crabs. This study underscores how food web stability is mediated by ecological interactions that can weaken or reinforce trophic cascades under variable natural conditions.

Introduction

Communities are structured through strong biotic interactions, including top-down effects of predation [1,2]. Top predators may stabilize basal prey populations (i.e., decrease chances of basal prey extirpation) by suppressing the predation interaction between intermediate predators and basal prey within simple tri-trophic cascades, ~~as defined by a single top predator, a single intermediate predator, and the basal prey~~ [3,4,5,6]. However, the stability of trophic cascades become less predictable as other interacting species are added [7]. Whether trophic cascades are destabilized by interactions among multiple predator species is a recent topic of intensive ecological interest [8,9].

Top predators indirectly enhance abundances of basal prey within trophic cascades [10]. Basal prey indirectly benefit from top predators both through the consumption of intermediate predators, or density-mediated indirect interactions (DMIIs) [11,12,13,14], as well as through suppression of feeding by intermediate predators, or trait-mediated indirect interactions (TMIIs) [3,5,6]. Resulting non-consumptive effects (NCEs) arise from non-lethal changes in biology or behavior leading to reduced fitness of intermediate predators [13,15,16]. For example, reduced feeding due to predator avoidance behavior will engender less energy for growth or reproduction [14,17].

Facilitative and competitive interactions among multiple predators within a community potentially result in non-additive effects on basal prey known as Multiple Predator Effects (MPEs) [18,19]. Synergistic MPEs cannot be predicted from effects of single predators in

isolation [14,20]. Moreover, interactions among multiple predators may stabilize or destabilize trophic cascades by reducing or increasing basal prey mortality [21]. Facilitation between top predators can destabilize trophic cascades through accelerated feeding on a basal resource [19]. Conversely, interference or predation between intraguild predators (i.e., species feeding on a common prey) [14,22] or size classes can suppress feeding on a common basal resource [23]. Thus, multiple predator species need to be considered together to discern their combined effects on shared basal prey [9].

In addition to species identity, trophic cascade stability should be responsive to other traits that mediate the risk of mortality to intermediate predators [24]. Body size is an overarching trait often overlooked within food webs [25]. For example, inhibitive effects of top predators on feeding behavior may vary with intermediate predator size, because predation risk decreases with the predator-prey size ratio [22]. Consequently, TMII strength should vary with the size ratio between top and intermediate predators [26]. Furthermore, MPE interactions may cause changes in the size selection of basal prey [20]. Moreover, interference or predatory interactions among intraguild members are strongly mediated by body size, which adds a horizontal dimension within food webs [23].

Habitat complexity can also mediate trophic cascades by weakening or reinforcing predation and interference interactions [14,27,28,29]. Structurally complex habitat potentially reduces predator efficiency by providing refuge for basal prey as well as for intermediate predators as prey [30,31,32,33,34]. Structurally complex habitats can stabilize trophic cascades by reducing predation on basal prey [17,35,36]. Conversely, interference may be mitigated within complex habitats by lowering encounter frequencies between intraguild predators

[22,29,36,37,38]. Thus, the degree to which trophic cascades are weakened or reinforced by habitat complexity also depends on the identities and body sizes of MPEs within any given system [9].

The oyster reef complex offers an ideal model system for the study of trophic interactions surrounding basal oyster prey [9,14,19,22,23]. Core assemblages comprising multiple species and sizes of coexisting top and intermediate predators can be readily manipulated [35]. Interference, predation and facilitation within assemblages can alter abundances of juvenile oysters in unpredictable ways [19]. Trophic interactions in turn are mediated by habitat complexity [14]. Previous studies of oyster reef trophic cascades demonstrate substantial context dependence in terms of predator identity [9,19], predator size [22,23] and habitat complexity [14,22].

Experimental studies of oyster reefs typically examine subsets of predators forming tri-trophic cascades [sensu 17] or triangular food web compartments [sensu 19], within which effects of competitive, predatory, or facilitative interactions influence cascade stability [39]. A classic tri-trophic cascade within the oyster-reef complex comprises the oyster toadfish (*Opsanus tau*), the mud crab (*Panopeus* spp.), and the juvenile oyster (*Crassostrea virginica*). Many studies show that oyster toadfish stabilize the trophic cascade by suppressing mud crab feeding on juvenile oyster [but see 40]. How additional predator species interact may further stabilize or destabilize the tri-trophic cascade [14].

A common experimental approach to detecting MPEs involves manipulating combinations of different types and sizes of predators [22,23]. However, potential effects of complex interactions have not been examined for the MPE configuration involving the toadfish,

the stone crab, and the mud crab. As top predators, toadfish consume mud crabs and juvenile stone crabs, but not oysters [41]. Stone crabs are intraguild predators [sensu 42] of mud crabs [9], and mud crabs potentially interfere with [14] or consume each other [23]. Both generalist stone crabs and mud crabs consume juvenile oysters [43].

The classic tri-trophic cascade may be stabilized or de-stabilized in the context of different types and sizes of predators. In addition, trophic interactions are mediated by habitat complexity. Here we focus on several potentially destabilizing/stabilizing effects on the classic toadfish-mud crab-oyster tri-trophic cascade, including addition of the intraguild Gulf stone crab (*Menippe adina*), habitat complexity as represented by oyster shell vs. limestone gravel substrates, and TMII effects posed by toadfish vs. stone crab relative to mud crab size. To examine these effects, two sets of laboratory experiments were conducted. Effects of predator configuration and habitat complexity were examined through mesocosm trials comprising exclusive combinations of Gulf toadfish (*Opsanus beta*), Gulf stone crab (*Menippe adina*), mud crab (*Panopeus herbstii*), two size classes of juvenile oysters, and two substrate types (oyster shell vs. limestone). TMII effects attributable strictly to avoidance behavior were examined through another set of experiments involving caged toadfish or stone crabs as top predators and different sizes of mud crabs as intermediate predators.

Materials and methods

Collection of organisms

Organisms were obtained using plastic sampling trays (52 cm x 52 cm x 11 cm) lined with self-closing mosquito netting (0.5 mm mesh) and filled with oyster shell (≈ 6,000 g). Trays

were deployed *in situ* for 10 weeks at various nearshore subtidal reefs along the Mississippi coast [44]. Live organisms for experiments were identified, measured, and placed into holding tanks. Test organisms were acclimated at 25°C and 29 ppt for at least three days. Individual crabs were kept isolated to prevent aggressive interactions. Reared juvenile oyster were obtained from the University of Auburn Shellfish Laboratory in Dauphin Island, AL for experiments. This study was carried out in strict accordance with the recommendations in the Guide for the Care and Use of Laboratory Animals of the National Institutes of Health. The protocol for this study was approved by the Institutional Animal Care and Use Committee (IUCUC) of the University of Southern Mississippi (USM) (Protocol Number: 16052606. All efforts to obtain, handle and house fish were made humanely. Following the completion of experiments, fish subjects were released back into the wild.

MPE experiment

Oyster survival was examined in response to different combinations of top and intermediate predators, and the two substrate types. Substrate types included either oyster shell or #57 limestone gravel, which is commonly used for oyster reef restoration. Substrate was soaked and cleaned to remove organisms or organic debris before using. Small mesocosms (89 x 48 x 33 cm tubs) filled with 110 l filtered seawater served as experimental units. One hour prior to the introduction of test organisms, 30 loose juvenile oysters (fifteen small (≈15 mm spat) and fifteen large (≈30 mm seed) were randomly distributed over the substrate. Heaters maintained the water temperature at 25° C, aerators provided water circulation and oxygenation. Abiotic variables were monitored with a handheld YSI model #85/25. Sizes of organisms were standardized: mud crabs (MC) ≈ 23 mm CW, stone crabs (SC) ≈ 60 mm CW, and

toadfish (TF) ≈ 120 mm total length; and emulated the most common sizes sampled from source reefs. Oyster prey densities ensured total prey depletion did not occur within 24 h. Four randomized blocks including all MPE treatment combinations were run separately for each substrate type: (1) 30 oyster (control); (2) seven mud crab (MC); (3) seven MC and one toadfish (TF); (4) seven MC and one stone crab (SC); (5) one SC; (6) one SC and one TF; (7) seven MC, one TF, and one SC. The TF/oyster treatment was not included, as toadfish do not consume oysters [40, 41]. Runs of each block ensued for 24 h, after which surviving numbers of oysters and mud crabs were enumerated. Test organisms were not reused to ensure independence.

TMII experiments

NCE proportions derived from the MPE experiment implied reduced mortality of basal prey due either to avoidance or interference behavior. Trait mediated indirect interaction (TMII) experiments focused on differences in juvenile oyster survival due strictly to avoidance behavior of different sizes of intermediate mud crab predators in the presence of caged predators or empty cage controls. Tanks (76 x 33 x 33 cm) filled with 75 l of filtered seawater at 29 ppt served as experimental units. Heaters regulated the water temperature at 25° C and overhead LED lights were kept on a 12-h light cycle. To isolate TMII effects, predators were retained within cylindrical (18 cm diameter x 30.5 cm tall) 2.6 cm mesh wire cages. Ten loose 15 mm oyster were distributed within each unit prior to the introduction of individual mud crabs belonging to one of three designated size classes: small (13-15 mm CW), medium (21-23 mm CW), or large (28-31 mm CW). The use of individual mud crabs precluded intraspecific interference. Empty cage treatments controlled for cage effects. Substrate was not used to strictly isolate behavioral responses to the presence of caged predators. Four randomized

blocks of six experimental units comprised three units containing individual mud crabs of each size-class in the presence of a caged predator (i.e., stone crab ≈ 65 mm carapace width; toadfish ≈ 120 mm TL), in addition to three control units containing individual mud crabs of each size-class and empty cages. The duration of each run was 24 h, after which the number of surviving oysters was enumerated. All runs for each type of top predator ensued over consecutive 4 d periods.

Another TMII experiment focused on interference vs. facilitation interactions between a large (28-30 mm CW mud crab) and three small intermediate predators (≈ 14-15 mm CW mud crabs), exposed either to caged toadfish (120 mm TL) or to empty control cages. Experimental conditions were identical to those for individual mud crab TMII experiments. Runs ensued for 24 h, with replacement of consumed oysters after 12 h due to higher feeding rates in this TMII experiment. Surviving oysters were enumerated at 12 and 24 h.

Statistical analysis

For mesocosm experiments, the mean number of oysters consumed and the mean number of mud crab consumed were responses within separate two-way ANOVAs comprising MPE Treatment and Substrate as crossed fixed factors using the General Linear Models procedure in SPSS (version 25). Both small and large oysters were pooled as a single response within the ANOVA, as survival of both size classes was strongly positively correlated ($r = 0.825$; $P_{1t} < 0.001$), and there was no discernable pattern in numbers of large vs. small oysters consumed among the seven MPE treatment levels. Paired t-tests between numbers of small and large oysters consumed were not different for every MPE treatment, when using the Holm-Bonferroni sequential correction method (i.e., effective rejection at $\alpha < 0.05$) [45]. The control

MPE treatment level (i.e., oyster alone) was omitted from the ANOVA, to avoid an omnibus test driven by the lack of oyster mortality observed for this treatment. Levene's and F-tests for equality of error variances and heteroscedasticity were not different for oyster mortality within the ANOVA. The ANOVA for the mean number of mud crabs consumed comprised the four MPE treatment levels that included mud crabs as an MPE species. Because the error variance was heterogeneous, the mud crab omnibus ANOVA was also tested with a two-way PERMANOVA using Manly's approach —Unrestricted Permutation of Observations in R. Differences in responses were compared among levels of the MPE Treatment factor and for the interaction of MPE Treatment by Substrate using Least Significant Difference (LSD) tests. Rejection levels for post-hoc comparisons among more than three levels were adjusted using the Holm-Bonferroni sequential correction method (i.e., rejection criterion at $\alpha < 0.05$) [45].

For the TMII experiment, the number of oysters consumed was analyzed with a two-way ANOVA including the two main factors, Predator (3 levels, stone crab (n=12), toadfish (n=12), No predator (n = 24), and Mud Crab Size (3 levels, small, medium, large) using the Generalized Linear Model (GLM) procedure within SPSS (version 25). Because the variance in oysters consumed was heterogeneous, omnibus tests were confirmed using a two-way PERMANOVA based on Manly's approach — Unrestricted Permutation of Observations in R. Differences among levels of main factors and their interaction were compared using Least Significant Difference (LSD) tests. An independent two-sample t-test in SPSS (version 25) compared numbers of oysters consumed between the two groups (toadfish present vs. toadfish absent) for the mixed-size intermediate predator (i.e., 1 large and 3 small mud crabs) TMII experiment. Variance was homogeneous between the two groups (F = 0.866; P = 0.388).

The possibility of risk reduction or enhancement with respect to oyster and mud crab survival was tested for various treatments from the MPE experiment using one-sample t-tests, following a multiplicative model approach as defined by Soluk and Collins and Sih et al. [15,18]. Following Grabowski et al. [14], expected oyster mortality values were adjusted for numbers of mud crabs consumed (i.e., 1.464 oysters per individual mud crab based on ½ the number of mud crabs consumed), to adjust for oysters not consumed by predated mud crabs. No such adjustment was needed for mud crab as prey, as no cannibalism by mud crabs was observed. Toadfish predation was assumed to be zero for calculations of expected numbers of oyster consumed. One-tailed t-tests reflected expected directionality in the outcomes, as indicated by the relative difference between expected and observed values. For the MPE combination involving all three predators (i.e., TF-SC-MC), two one-sample t-tests were based on different expected values generated using either the three single predator outcomes or the two top predator and mud crab combinations. For the MPE combination involving all three predators (i.e., TF-SC-MC) for which mud crabs were prey, two one-sample t-tests were based on different expected values generated either using single stone crab and toadfish outcomes from both substrates, or expected values based on only limestone treatments - as there was a strong interaction with substrate. Where risk reduction was inferred by observed values that were less than expected values, consumptive effect (CE) and non-consumptive effect (NCE) proportions were calculated following Grabowski et al. [14].

For the MPE experiment, NCE proportions implied reduced mortality of basal prey due either to avoidance behavior and/or to predator interference [14]. NCE proportions for TMII experiments were obtained directly from the difference in oyster mortality between caged

predator and empty cage treatments. Non-consumptive effect proportions from the TMII experiment strictly reflected effects of avoidance behavior by different sizes of individual mud crabs in the absence of interference. In contrast, the NCE proportion calculated for the TMII experiment based on mixed sizes of mud crabs reflected interference or facilitative interactions, in addition to avoidance behavior.

Results

MPE Experiment

Both MPE Treatment and Substrate factors varied meaningfully relative to the total number of oysters consumed within the two-way ANOVA, as well as to the interaction between the two main factors (Table 1). The mean number of oysters consumed was higher in limestone (15.6 ± 1.4 se) than in shell (11.3 ± 1.4 se). Numbers of oysters consumed shifted toward higher loss in limestone across all MPE treatment levels, except for the TF/MC treatment, for which the number of oysters consumed by mud crabs was five-fold higher in shell than in limestone ($t = 3.14$; $P_{1\text{-}t} = 0.007$; log scale) (Table 2; Figure 1). Mean numbers of oysters consumed across both substrate types ranged from 3.7 for the TF/MC treatment to 20.5 for the MC treatment (Table 2), yielding the largest difference of 16.8 oysters consumed. The TF/MC treatment was different (i.e., sequential Bonferroni adjusted LSD tests) from the MC ($P < 0.001$), TF/SC/MC ($P = 0.001$), TF/SC ($P = 0.003$), and SC/MP ($P = 0.003$) treatments. MPE risk-model t-tests implied a much stronger NCE when toadfish was the only top predator with mud crabs (0.81) than when only stone crabs were together with mud crabs (0.37), or when both stone crab and toadfish were together with mud crabs (0.25) (Table 3).

Table 1. Two-way ANOVA of the total number of oysters consumed within the MPE experiment (bold P ≤ 0.05).

Source	Type III Sum of Squares	df	Mean Square	F	P	Partial Eta Squared	Observed Power[b]
**Corrected Model**	2219.733[a]	11	201.794	3.557	**0.001**	0.449	.987
**Intercept**	10881.067	1	10881.067	191.793	**< 0.001**	0.800	1.000
**Substrate**	281.667	1	281.667	4.965	**0.031**	0.094	0.588
**MPE Treatment**	1547.333	5	309.467	5.455	**< 0.001**	0.362	0.982
**Substrate × Treatment**	390.733	5	78.147	1.377	0.249	0.125	0.444
**Error**	2723.200	48	56.733				
**Total**	15824.000	60					
**Corrected Total**	4942.933	59					

Table 2. Mean ± SD values for total number of oysters consumed for MPE treatment levels for shell and limestone substrata, as well as totals (maximum possible = 30) (MPE treatments: SC = stone crab; TF/SC = toadfish and stone crab; MC = mud crab; SC/MC = stone crab and mud crab; TF/MC = toadfish and mud crab; TF/SC/MC = toadfish, stone crab and mud crab).

Substrate	MPE Treat	Mean	Std. Deviation
**Shell**	**SC**	8.20	7.463
	**TF/SC**	8.40	3.050
	**MC**	17.80	5.541
	**SC/MC**	13.00	5.339
	**TF/MC**	6.20	4.324
	**TF/SC/MC**	14.20	9.311
	**Total**	11.30	6.964
**Limestone**	**SC**	16.20	9.602
	**TF/SC**	19.80	11.167
	**MC**	23.20	3.564
	**SC/MC**	15.20	10.756
	**TF/MC**	1.20	2.168
	**TF/SC/MC**	18.20	10.060
	**Total**	15.63	10.594
**Total**	**SC**	12.20	9.138
	**TF/SC**	14.10	9.780
	**MC**	20.50	5.233
	**SC/MC**	14.10	8.089
	**TF/MC**	3.70	4.165
	**TF/SC/MC**	16.20	9.378
	**Total**	13.47	9.153

Table 3. MPE risk-model t-tests. (Correction for Mud Crab (MC cor N) = 1.4643 oysters per individual MC lost; No SC/MC or TF/MC tests necessary or NCE applicable for mud crabs as prey because no MC cannibalism observed; toadfish predation on oysters assumed zero; TF+SC+MC_a – toadfish, stone crab and mud crab MPE relative to three single predator combinations; TF+SC+MC_b – toadfish, stone crab and mud crab MPE relative to two top predator and mud crab combinations; TF+SC+MC_c – stone crab stone crab and mud crab MPE from limestone treatments; TF+SC+MC_d – toadfish stone crab and mud crab MPE from both substrates; Negative t-values connote MPE risk reduction for basal prey; Positive t-values imply MPE risk enhancement for basal prey; 1-tailed t-tests reflect expected directionality in the outcomes, as indicated by whether observed values are greater or less than expected values) (bold P ≤ 0.05).

MPE	Prey type	Exp N	MC N	Obsv N	SE Obs	t-val	P_{1-t}	CE	NCE
TF+MC	Oyster	19.04	20.50	3.70	1.32	-11.645	**<0.001**	0.19	0.81
SC+MC	Oyster	24.36	22.46	14.10	2.56	-3.268	**0.005**	0.63	0.37
TF+SC	Oyster	12.20	NA	14.10	3.10	0.614	0.277	NA	NA
TF+SC+MC_a	Oyster	24.36	21.58	16.20	2.97	-1.814	**0.051**	0.75	0.25
TF+SC+MC_b	Oyster	16.06	13.28	16.20	2.97	0.985	0.175	NA	NA
TF+SC+MC_c	Mud crab	3.23	NA	3.80	0.58	0.980	0.191	NA	NA
TF+SC+MC_d	Mud crab	2.11	NA	1.90	0.69	-0.310	0.381	0.90	0.10

Table 4. Two-way ANOVA of the total number of mud crabs consumed within the MPE experiment (bold P ≤ 0.05).

Source	Type III Sum of Squares	df	Mean Square	F	P
**Corrected Model**	71.900[a]	7	10.271	9.130	**<0.001**
**Intercept**	44.100	1	44.100	39.200	**<0.001**
**Substrate**	25.600	1	25.600	22.756	**<0.001**
**MPE Treatment**	18.900	3	6.300	5.600	**0.003**
**Substrate × Treatment**	27.400	3	9.133	8.119	**<0.001**
**Error**	36.000	32	1.125		
**Total**	152.000	40			
**Corrected Total**	107.900	39			

a. R Squared = .666 (Adjusted R Squared = .593)

Both MPE Treatment and Substrate factors as well as their interaction varied strongly within the two-way ANOVA for the number of mud crabs consumed within the MPE experiment (Table 4). Because variance was heterogeneous (Levene Statistic = 6.465; $P < 0.001$), a two-way PERMANOVA was used to confirm main factor and interaction effects (MPE $P = 0.0026$; Substrate < 0.001; MPE × Substrate = 0.001). The interaction reflected non-parallel and higher mortality on mud crabs in limestone than oyster shell for the MPE treatments that included stone crabs (i.e., SC/MC and TF/SC/MC) vs. more equitable mortality across both substrate types for the TF/MC and MC treatments. The mean number of mud crabs lost to predation varied from 0 for the MC, to 1.0 for the TF/MC, 2.6 for the SC/MC, and 3.8 for the TF/SC/MC MPE treatment. Thus, more mud crabs were consumed in treatments involving stone crabs, and implied NCEs on oysters were weaker for stone crabs (0.37) than for toadfish (0.81) (Table 3). Post-hoc pairwise LSD tests of differences in mean numbers of mud crabs consumed showed that the MC treatment was different from both MPE treatments involving stone crabs ($P < 0.001$ for MC vs. SC/MC; $P = 0.01$ for MC vs. TF/SC/MC). The P value of 0.043 for MC vs. TF/MC treatment was nominally greater than 0.05 when adjusted using the sequential Bonferroni correction, but no mud crab cannibalism occurred within the MC treatment during the 24 h period. Mud crab mortality within the TF/SC/MC treatment was not very different from additive, whether considering both substrates together (exp N = 2.11 vs. obs N = 1.90) or just limestone (exp N = 3.23 vs. obs N = 3.80) (Table 3). Accordingly, a low NCE of 0.10 was apparent for mud crabs in the presence of both toadfish and stone crab (Table 3).

TMII Experiments

Both main factors, Predator and Mud Crab Size, as well as their interaction, differed for the number of oysters consumed within the two-way ANOVA of the TMII cage experiment (Table 5). Because variance was heterogeneous (Levene Statistic = 4.925; $P < 0.001$), an auxiliary two-way PERMANOVA was confirmed the main factor and interaction effects (predator $P < 0.001$; MC Size $P < 0.001$; predator × MC Size = 0.0034). The interaction effect reflected non-parallel declines in numbers of oysters consumed with crab size across the three predator levels (Figure 2). More oysters were consumed with increasing crab size within the empty cage treatment (LSD small < medium $P < 0.001$; LSD small < large $P < 0.001$; LSD medium < large P =0.012); whereas, oyster consumption did not vary appreciably with crab size within the caged stone crab or toadfish treatments (LSD P = 0.17 – 1.0). Neither did oyster consumption differ with crab size when data for both types of top predators were combined (LSD P = 0.18 – 0.87). Oyster consumption was roughly an order of magnitude higher in the absence of top predators; the mean number of oysters lost to predation by all size classes of mud crab was 4.87 ± 0.31 without top predators, vs. 0.58 ± 0.44 and 0.17 ± 0.44 in the presence of stone crab and toadfish, respectively. Oyster consumption was notably higher for the empty cage treatment than for either of the top predator treatments (LSD $P < 0.001$); and the top predator treatments did not differ from each other (LSD P = 0.504). Eight-fold fewer oysters were consumed in the presence of caged toadfish (4.5 ± 2.1) than when toadfish were absent (16.8 ± 3.3) (t = 3.165; P_{1t} = 0.0095) for the mixed-size mud crab TMII experiment.

Table 5. Two-Way ANOVA of total number oysters eaten relative to Predator (i.e., 3 levels = empty cage, Toadfish, or Stone crab) and Mud Crab Size (i.e, 3 levels = small, medium, and large) factors (bold P ≤ 0.05).

Source	Type III Sum of Squares	df	Mean Square	F	P
**Corrected Model**	373.875[a]	8	46.734	20.393	**<0.001**
**Intercept**	151.875	1	151.875	66.273	**<0.001**
**Predator**	244.041	2	122.021	53.245	**<0.001**
**MC Size**	45.000	2	22.500	9.818	**<0.001**
**Predator * MC Size**	44.958	4	11.240	4.905	**0.003**
**Error**	89.375	39	2.292		
**Total**	794.000	48			
**Corrected Total**	463.250	47			

R squared = 0.807 (Adjusted R Squared = 0.767)

Inferred NCEs due to behavioral suppression varied with mud crab size within the first TMII experiment, and were less pronounced for caged stone crab than for caged toadfish (Table 6). Large mud crabs showed the lowest NCE values, although NCE values were relatively high for all three size classes (i.e., all > 0.79). The NCE value for the mixed-size TMII experiment was lower than that for the individual large mud crab treatment from the first TMII experiment in the presence of toadfish (NCE = 0.73 vs. 0.93), implying interference among mud crabs did not markedly influence the consumption of oysters.

Table 6. Consumptive effects (CE) vs. non-consumptive effects (NCE = 1 – CE) for caged predator and mud crab size combinations in TMII experiments. (BOTH + data combined for both caged toadfish and stone crab; L, M, and S = large, medium, and small mud crab; L/S = mixed size mud crab TMII experiment.

Treatment	Predator Present	Predator Absent	CE	NCE
SC + MC_L	1.50	7.375	0.203	0.797
SC + MC_M	0.25	5.375	0.046	0.953
SC + MC_S	0.00	1.875	0.000	1.000
TF + MC_L	0.50	7.375	0.068	0.932
TF + MC_M	0.00	5.375	0.000	1.000
TF + MC_S	0.00	1.875	0.000	1.000
BOTH+MC_L	1.00	7.375	0.136	0.864
BOTH+MC_M	0.125	5.375	0.023	0.977
BOTH+MC_S	0.00	1.875	0.000	1.000
TF + $MC_{L/S}$	4.50	16.75	0.269	0.731

Discussion

Studies of MPEs typically focus on how interspecific interactions stabilize the trophic web through non-additive predator effects [15]. In addition to predator identity [9], other ecological factors may mediate the stability of oyster-reef trophic cascades including, body size [23], predator density [46], and habitat complexity or patchiness [14,47]. Thus, stability of a given oyster-reef food web is determined within the context of unique suites of mediating ecological factors [9,36]. Even given the tractable food web, considering all trophic interactions within the oyster-reef is unwieldy. Thus, experimental factors are selected judiciously in order to obtain new insights into oyster food-web stability. Here, we chose to examine the classic tri-trophic toadfish-mud crab-juvenile oyster cascade in connection with the interactive role played by the intraguild stone crab, a common resident of Gulf of Mexico (GoM) oyster reefs.

The presence of toadfish as a primary stabilizing influence on the tri-trophic cascade involving mud crab as the intermediate predator was evident in our study. However, intraspecific interference and cannibalism did not further stabilize the cascade, as in Geraldi [23]. Moreover, unlike Grabowski et al. [14], where the cascade was reinforced by interactions between toadfish and the generalist blue crab (i.e., capable of consuming both intermediate predators and basal prey), the addition of the stone crab as a generalist predator destabilized the tri-trophic cascade in our study. Like blue crabs, stone crabs consume both mud crabs and oysters, but toadfish did not inhibit stone crab feeding behavior in our study. Generalists feeding on both intermediate predators and basal prey have also been shown to destabilize oyster trophic cascades in other studies [48]. In addition to demonstrating both consumptive and non-consumptive effects on mud crabs, stone crabs also directly consumed oysters in the present study. However, NCEs relative to oyster consumption by mud crabs were less pronounced for stone crab than for toadfish. Thus, the primary tri-trophic effect was destabilized when all three predators were together, as opposed to when toadfish and mud crabs were the only two predators.

Weaker ~~implicit~~ NCEs relative to oyster consumption when all three predators were together than when toadfish and stone crabs occurred separately with mud crabs suggests interference between toadfish and stone crabs might contribute to destabilization of the trophic cascade. ~~Ironically,~~ direct predation by stone crabs on mud crabs was higher than for toadfish. Conversely, consumptive effects of mud crabs on oysters were higher in the presence of stone crabs for both MPE and TMII experiments. However, consumptive effects of toadfish and stone crabs on mud crabs appeared additive when all three predators were together. ~~And~~

the effect of toadfish on mud crab consumption by stone crabs was lacking. Nor did toadfish suppress feeding by stone crabs on oysters in the absence of mud crabs. As illustrated by our TMII experiment, toadfish elicited greater NCEs on oyster consumption by mud crabs than did stone crabs. Other studies show that toadfish induce mud crabs to seek refuge and limit their movements [14]. Stone crabs are more adept at flushing mud crabs from refuges, whereas toadfish are ambush predators [47]. Consequently, higher encounter rates between mud crabs and toadfish as well as oysters might occur when all three predators are together. Depending on prey size, stone crabs may prefer mud crabs as a more profitable prey type when oysters are also available, but this needs to be tested.

It would not have been unexpected for toadfish to suppress stone crab feeding behavior, because juvenile stone crabs are common residents of GoM oyster reefs and regularly occur in the diets of toadfish [41]. However, stone crab feeding was not suppressed by toadfish in our MPE experiment. The sizes of stone crabs used in our MPE experiment matched the most common size class found on source reefs. Although the total lengths of MPE toadfish (i.e., 120 mm TL) were about two fold higher than the carapace widths of MPE stone crabs, stone crabs may have been ontogenetically too large to perceive much risk of toadfish predation. However, toadfish can grow large enough (i.e., > 300 mm TL) [23] to pose a risk to adult stone crabs, making stone crabs of the sizes used in our experiments potentially vulnerable to predation by toadfish in the field.

Body size is an important ecological factor mediating the stability of food webs through predator-prey interactions between top and intermediate predators [49] and between intermediate or generalist (i.e., multi-level) predators and basal prey [20,43,48]. Body size also

mediates interference competition or predation among intraguild predators [22,23]. For example, size-selection of hard clams (*Mercenaria*) proved important for understanding MPEs relative to blue crabs' preference for small clams, but was not important for stone crabs. Except for some bias for large oysters (i.e., 30 mm seed) by stone crab and for small oysters (i.e., 15 mm spat) by mud crab, no definitive pattern in the size-selection of oysters emerged in our MPE experiment. Paired t-tests between the two oyster-size classes were not different across all MPE treatments after correction for multiple testing, confirming all types of predators were capable of consuming both sizes of oysters in our MPE experiment. When considered over a wide enough range of sizes (i.e., up to 70+ mm VLV), costs associated with handling time or crushing strength of hard shell molluscan prey has been shown to increase with predator/prey size, and potentially affect feeding rates of stone crabs, blue crabs, and mud crabs, [43]. Oysters below a size threshold of 25 mm has been shown to separate smaller oysters within a size refuge from stone crabs; however their small size makes them vulnerable to mud crabs. Rindone and Eggleston [43] used larger stone crabs (80-130 mm CW) than those in our MPE experiment (60 mm CW). And mud crabs (23 mm CW) in our MPE experiment consumed oysters in both size classes. Indeed, both stone crabs and mud crabs possess relatively strong crushing chelae.

Risk to intermediate predators is also mediated by body size [22]. Accordingly, we observed size-related NCE release for mud crabs with respect to the two top predators. Plasticity in the expression of TMIIs is an important influence on trophic cascades [13]. Different sizes of intermediate predators should respond commensurately with the threat of predation, as a balance between risk and energetic trade-offs [6]. Thus, TMII strength should vary with the

size ratio of top to intermediate predator [26], and the size structure of the intermediate predator population will mediate predation pressure on basal prey populations. Our finding of size-related NCE release for mud crabs compliments Geraldi [23], who showed that the trophic cascade was expressed differently with respect to size distributions and densities of intermediate mud crabs. However, in contrast to our study, Geraldi [23] noted that the trophic cascade was stabilized by interference and cannibalism among mud crabs. Moreover, mud crab interactions exerted as much influence on stability as did the top predator on NCEs. Neither interference nor cannibalism occurred among mud crabs in our study. However, a much wider size range of mud crabs was used over a longer time period by Geraldi [23]. However, cannibalism mostly occurred among smaller mud crabs, likely due to an inverse relationship between molting frequency (i.e., vulnerability) and crab size. The shorter time period of our MPE experiment precluded molting frequency as a contributing factor. Additionally, density-induced interference among mud crabs was likely encouraged by representing different sizes of organisms in terms of biomass equivalents in the Geraldi study [23]. Using biomass equivalents of oyster reef predators typically shows that predation on oysters is density mediated, as small-bodied mud crabs are represented by many individuals per unit biomass [14,23]. Accordingly, the exclusion of mud crabs by mesh enclosures has been shown to promote oyster survival in the field [40,48]. Thus, interference and cannibalism among mud crabs would stabilize the cascade. In contrast, size-related mud crab NCE release observed in our study is potentially destabilizing. Furthermore, NCEs were not noticeably strengthened by interference among mud crabs of mixed sizes in our TMII experiment. Clearly, size structures of populations of both

predators and prey are important in the expression of density mediated and trait mediated indirect interactions within oyster-reef food webs.

Habitat complexity can affect the stability of oyster-reef trophic cascades through the provision of refuge, or by relaxing predation on basal prey [14] and intermediate predators [22], or by dampening interference [29,36]. Oyster-shell exhibits greater habitat complexity than limestone gravel, due to its profuse three-dimensional structure comprising many crevices. Surface-area to volume ratios of exposed oyster shell average three to nine fold higher than exposed limestone under field conditions [50]. In our study, loose juvenile oysters survived better in oyster shell than in limestone substrate, reflecting the general stabilizing effect of complex habitat [14]. However, a destabilizing aspect of habitat complexity on the tri-trophic cascade was also revealed by higher consumption of oysters by mud crabs in oyster shell than in limestone, when in the presence of toadfish. Presumably, higher encounter rates between mud crabs and oysters was facilitated by more active feeding of mud crabs within complex shell substrate. Thus, the stabilizing effect of toadfish within the classic tri-trophic cascade was weakened to some extent by habitat complexity. In contrast, Grabowski et al. [14] found that the cascade was stabilized when mud crabs utilized refuge and were thus less active in the presence of both toadfish and blue crabs. However other studies have also found that habitat complexity can destabilize the tri-trophic cascade by reducing interference among and between intermediate or top predators [29,36]. Thus like for other factors, the extent to which habitat complexity stabilizes the oyster trophic cascade is context dependent. Effects of habitat complexity are particularly important to consider in the light of oyster-reef restoration efforts, which often use limestone gravel as cultch [50,51,52,53].

Although our mesocosm experiments emulated oyster reef habitat, they differed from natural conditions in several notable respects. Selected taxa represented a small subset of the oyster reef community. Consequently, predators were precluded from having mixed diet choices [14]. Although densities of predators were comparable to the field, they did not vary across treatments. Temperature and salinity were maintained at levels similar to field conditions, but did not fluctuate. The use of loose spat likely affected predation efficiency in terms of handling. Cultchless oysters have been shown to have lower survival than attached oysters [40]. Although two size classes of oyster prey were presented, the prey size distribution was not fully represented. Experiments also incurred some prey depletion, although full depletion was precluded by experimental time periods based on pilot runs. Tank effects were also possible. Experimental units potentially restricted predator movements, affected predation efficiency, and increased chances of predator interference. Isolation of experimental units potentially enhanced NCEs by concentrating chemical cues relative to field conditions [46]. However, isolated conditions minimized chances of misconstruing predator avoidance effects. Stronger NCEs in TMII experiments possibly reflected the lack of refuge normally provided by natural substrate, while also ruling out substrate as an extraneous factor. Thus, the TMII experiments revealed that NCEs were strictly due to the suppression of mud crab behavior. By sacrificing realism, laboratory experiments facilitate tests of specific hypotheses about mechanisms operating in nature.

Conclusion

Notwithstanding possible artifacts, our experiments revealed several potential sources of destabilization of a classic tri-trophic cascade that compliment previous studies. First, the

generalist stone crab consumed oysters and mud crabs undeterred by the presence of toadfish. Feeding on oysters by mud crabs was suppressed by the presence of either stone crabs or toadfish, but considerably less so by stone crabs. Consequently, the sum total effect of all three predator species together was destabilizing, because considerably more oysters were consumed than when toadfish and mud crabs were the only two predators. While NCEs relative to mud crab feeding on oyster as prey were expressed in the presence of all three predators, they were weaker than for each top predator by itself. Conversely, weaker inferred avoidance by mud crabs in the presence of stone crabs resulted in culminated in relatively high direct predation on mud crabs. TMII experiments confirmed stronger avoidance of mud crab to toadfish than to stone crabs, and revealed that NCEs with respect to oyster prey in the MPE experiment were mostly due to behavioral suppression of mud crabs, rather than to interference by other mud crabs or top predators. The inverse relationship between mud crab body size and NCE strength inferred by the TMII experiment potentially contributes an additional destabilizing influence on the tri-trophic-cascade. Finally, although habitat complexity generally stabilized the consumption of oysters across MPE treatments, greater habitat complexity destabilized the tri-trophic cascade itself, by relaxing suppression of feeding by mud crabs. Nevertheless, the toadfish-mud crab-oyster configuration was the most effective MPE treatment for minimizing oyster consumption, regardless of substrate type. Although multiple studies have confirmed the existence of the tri-trophic cascade, some have questioned its relevance under field conditions [40]. This study underscores how the predictability of food web stability can be obscured by mediating ecological interactions that can weaken or reinforce trophic cascades under variable natural conditions

Acknowledgements

Many thanks are due to Scott Rikard and the Auburn Shellfish Laboratory at Dauphin Island for providing oyster spat for this study. We also want to thank the USM GCRL staff, especially Chris Lapniewski and Scott McIntosh, who helped with field work.

Author Contributions

Conceptualization: Virginia R. Fleer, Chet F. Rakocinski.

Data curation: Virginia R. Fleer.

Formal analysis: Chet F. Rakocinski.

Funding acquisition: Virginia R. Fleer, Chet F. Rakocinski.

Investigation: Virginia R. Fleer.

Methodology: Virginia R. Fleer, Chet F. Rakocinski.

Project administration: Virginia R. Fleer, Chet F. Rakocinski.

Resources: Virginia R. Fleer, Chet F. Rakocinski.

Supervision: Virginia R. Fleer, Chet F. Rakocinski.

Validation: Virginia R. Fleer.

Visualization: Chet F. Rakocinski.

Writing – original draft: Virginia R. Fleer.

Writing – review & editing: Chet F. Rakocinski, Virginia R. Fleer.

References

1. Paine RT. Food web complexity and species diversity. *The American Naturalist*. 1966; 100: 65-75.
2. Paine RT. Intertidal community structure. Experimental studies on the relationship between a dominant competitor and its principal predator. *Oecologia*. 1974; 15: 93-120.
3. Schmitt, RJ. Indirect Interactions between prey: Apparent competition, predator aggregation, and habit segregation. *Ecology*. 1987; 68: 1887-1897. doi:10.2307/1939880.
4. McQueen DJ, France R, Post JR, Stewart TJ, Lean DRS. Bottom-up and top-down impacts on freshwater pelagic community structure. *Ecological Monographs*. 1989; 59: 289–309.
5. Wootton, JT. 1993. Indirect effects and habitat use in an intertidal community: Interaction chains and interaction modifications. *The American Naturalist*. 1993; 141: 71. doi:10.1086/285461.
6. Werner EE, Peacor SD. 2003. A Review of trait-mediated indirect interactions in ecological communities. *Ecology*. 2003; 84: 1083–1100. doi:10.1890/0012-9658(2003)084[1083:AROTII]2.0.CO;2.
7. Grason EW, Miner BG. (2012) Preference alters consumptive effects of predators: Top-down effects of a native crab on a system of native and introduced prey. PLoS ONE. 2012; 7(12): e51322. doi:10.1371/journal.pone.0051322.
8. Bruno JF, O’Connor MI. Cascading effects of predator diversity and omnivory in a marine food web. *Ecology Letters*. 2005; 8: 1048–56. doi:10.1111/j.1461-0248.2005.00808.x.

9. O'Connor, NE, Grabowski, JH, Ladwig, LM, Bruno, JF. Simulated predator extinctions: Predator identity affects survival and recruitment of oysters. *Ecology*. 2008; 89: 428–38. doi:10.1890/06-2029.1.

10. Schmitz, OJ. Direct and indirect effects of predation and predation risk in old-field interaction webs. *The American Naturalist*. 1998; 151: 327-342.

11. Carpenter, SR, Kitchell, JF, Hodgson, JR. Cascading trophic interactions and lake productivity. *BioScience*. 1985; 35: 634–39. doi:10.2307/1309989.

12. Strong, DR. Are trophic cascades all wet? Differentiation and donor-control in speciose ecosystems. *Ecology*. 1992; 73: 747-754. doi:10.2307/1940154.

13. Schmitz, OJ, Krivan, V, Ovadia, O. Trophic Cascades: The primacy of trait-mediated indirect interactions. *Ecology Letters*. 2004; 7: 153-163.. doi:10.1111/j.1461-0248.2003.00560.x.

14. Grabowski, JH, Hughes, AR, Kimbro, DL. Habitat complexity influences cascading effects of multiple predators. *Ecology*. 2008; 89: 3413–22. doi:10.1890/07-1057.1.

15. Sih, A, Bolnick, DI, Luttbeg, B, Orrock, JL, Peacor, SD, Pintor, LM, Preisser, E, Rehage, JS, Vonesh, JR. Predator-prey naïveté, antipredator behavior, and the ecology of predator invasions. *Oikos*. 2010; 119: 610–621.

16. Hermann, SL, Landis, DA. Scaling up our understanding of non-consumptive effects in insect systems. *Current Opinion in Insect Science*. 2017; 20: 54–60.

17. Grabowski, JH. Habitat complexity disrupts predator-prey Interactions but not the trophic cascade on oyster reefs. *Ecology*. 2004; 85: 995–1004.

18. Soluk, DA, Collins, NC. Synergistic Interactions between fish and stoneflies: Facilitation and interference among stream predators. *Oikos*. 1988; 52: 94–100. doi:10.2307/3565987.

19. Fodrie, FJ, Kenworthy, MD, Powers, SP. Unintended facilitation between marine consumers generates enhanced mortality for their shared prey. *Ecology*. 2008; 89: 3268–3274.

20. Wong, MC, Peterson, CH, Kay, J. Prey size selection and bottom type influence multiple predator effects in a crab-bivalve system. *Marine Ecology Progress Series*. 2010; 409: 143–156. https://doi.org/10.3354/meps08621

21. Crowder, LB, Squires, DD, Rice, JA. Nonadditive effects of terrestrial and aquatic predators on juvenile estuarine fish. *Ecology*. 1997; 78: 1796–1804. https://doi.org/10.1890/0012-9658(1997)078[1796:NEOTAA]2.0.CO;2

22. Hill, JM, Weissburg, MJ. Habitat complexity and predator size mediate interactions between intraguild blue crab predators and mud crab prey in oyster reefs. *Marine Ecology Progress Series*. 2013; 488: 209–19. doi:10.3354/meps10386.

23. Geraldi, NR. Prey size structure diminishes cascading effects by increasing interference competition and predation among prey. *Ecology*. 2015; 96: 2533–2543. https://doi.org/10.1890/14-1026.1

24. Abrams, PA. Implications of dynamically variable traits for identifying, classifying and measuring direct and indirect effects in ecological communities. *The American Naturalist*. 1995; 146: 112–134.

25. Raffaelli D. Food webs, body size and the curse of the Latin binomial. In: Rooney N, McCann KS, Noakes DLG. (eds) *From Energetics to Ecosystems: The Dynamics and Structure of Ecological Systems.*2007; Springer, Dordrecht.

26. Freeman, A. Size-dependent trait-mediated indirect interactions among sea urchin herbivores. *Behavioral Ecology*. 2006; 17: 182–187. https://doi.org/10.1093/beheco/arj014

27. Murdoch, WW, Oaten, A. 1975. Predation and population stability. *Advances in Ecological Research*. 1975; 9: 1–131. doi:10.1016/S0065-2504(08)60288-3.

28. Crowder, LB, Cooper, WE. Habitat stuctural complexity and the interaction between bluegills and their prey (*Lepomis macrochirus*). *Ecology*. 1982; 63: 1802-1813. doi:10.2307/1940122.

29. Grabowski, JH, Powers, SP. Habitat complexity mitigates trophic transfer on oyster reefs. *Marine Ecology Progress Series*. 2004; 277: 291–95. doi:10.3354/meps277291.

30. Pace, ML, Cole, JJ, Carpenter, SR, Kitchell, JF. Trophic cascades revealed in diverse ecosystems. *Trends in Ecology and Evolution*. 1999; 14: 483-488. doi:10.1016/S0169-5347(99)01723-1.

31. Littler, MM, Taylor, PR, Littler, DS. Complex Interactions in the control of coral zonation on a Caribbean reef flat. *Oecologia*. 1989; 80 (3): 331–40. doi:10.1007/BF00379034.

32. Diehl, S. Fish predation and benthic community structure: The role of omnivory and habitat complexity. *Ecology*. 1992; 73: 1646-1661. doi:10.2307/1940017.

33. Schriver P, Bøgestrand, J , Jeppesen, E, Søndergaard, M. Impact of submerged macrophytes on fish-zooplankton-phytoplankton interactions. *Freshwater Biology*. 1995; 33: 255-270.

34. Beukers, JS, Jones, GP. Habitat complexity modifes the impact of piscivores on a coral reef fish population. *Oecologia*. 1997; 114: 50–59. doi:10.1007/s004420050419.

35. Grabowski, JH, Hughes, AR, Kimbro, DL, Dolan, MA. How habitat setting influences restored oyster reef communities. *Ecology*. 2005; 86: 1926–35. doi:10.1890/04-0690.

36. Hughes, AR, Grabowski, JH. Habitat context influences predator interference interactions and the strength of resource partitioning. *Oecologia*. 2006; 149: 256–64. doi:10.1007/s00442-006-0439-7.

37. Swisher, BJ, Soluk, DA, Wahl, DH. Non- additive predation in littoral habitats: influences of habitat complexity. *Oikos*. 1998; 81:30–37.

38. Griffen, BD, Byers, JE. Partitioning mechanisms of predator interference in different habitats. *Oecologia*. 2006; 146: 608–14. doi:10.1007/s00442-005-0211-4.

39. Krause, AE, Frank, KA, Mason, DM, Ulanowicz, RE, Taylor, WW. Compartments revealed in food-web structure. *Nature*. 2003; 426: 282–285. https://doi.org/10.1038/nature02115

40. Abbe, GR, Breitburg, DL. The influence of oyster toadfish (*Opsanus tau*) and crabs (*Callinectes sapidus* and Xanthidae) on survival of oyster (*Crassostrea virginica*) spat in Chesapeake Bay: Does spat protection always work? *Aquaculture*. 1992; 107: 21–31. doi:10.1016/0044-8486(92)90047-O.

41. Shervette, VR, Perry, HM, Rakocinski, CF, Biesiot, PM. Factors influencing refuge occupation by stone crab *Menippe adina* juveniles in Mississippi Sound. *Journal of Crustacean Biology*. 2004; 24: 652–665.

42. Ives, AR, Cardinale, BJ, Snyder, WE. A synthesis of subdisciplines: predator–prey interactions, and biodiversity and ecosystem functioning. *Ecology Letters*. 2005; 8: 102-116.

43. Rindone, RR, Eggleston, DB. Predator-prey dynamics between recently established stone crabs (*Menippe* spp.) and oyster prey (*Crassostrea virginica*). *Journal of Experimental Marine Biology and Ecology*. 2011; 407: 216–225. https://doi.org/10.1016/j.jembe.2011.06.018

44. Fleer, VR. *Effects of Trophic Relationships on Oyster Reef Restoration Success in the Mississippi Sound*. 2017; (Doctoral Dissertation) University of Southern Mississippi, Hattiesburg.

45. Holm, S. A simple sequentially rejective multiple test procedure. *Scandinavian Journal of Statistics*. 1979; 6: 65–70.

46. Hughes, AR, Rooker, K, Murdock, M, Kimbro, DL. Predator cue and prey density interactively influence indirect effects on basal resources in intertidal oyster reefs. *PLoS ONE*. 2012; 7: e44839. doi:10.1371/journal.pone.0044839.

47. Macreadie, PI, Geraldi, NR, Peterson, CH. Preference for feeding at habitat edges declines among juvenile blue crabs as oyster reef patchiness increases and predation risk grows. *Marine Ecology Progress Series*. 2012; 466: 145–153. https://doi.org/10.3354/meps09986

48. Johnson, KD, Grabowski, JH, Smee, DL. Omnivory dampens trophic cascades in estuarine communities. *Marine Ecology Progress Series*. 2014; 507: 197–206. https://doi.org/10.3354/meps10815

49. Schneider, FD, Scheu, S, Brose, U. Body mass constraints on feeding rates determine the consequences of predator loss. *Ecology Letters*. 2012; 15: 436–443. https://doi.org/10.1111/j.1461-0248.2012.01750.x

50. KuyKendall, KM, Moreno, P, Powell, EN, Soniat, TM, Colley, S, Mann, R, Munroe, DM. The exposed surface area to volume ratio: Is shell more efficient than limestone in promoting oyster recruitment? *Journal of Shellfish Research*. 2015; 34: 217–225.

51. Hidu, H, Chapman, S, Soule, PW. Cultchless setting of European oysters *Ostrea-edulis* using polished marble. *Proceedings National Shellfisheries Association*. 1974; 65: 13–14.

52. Soniat, TM, Broadhurst, BC III, Haywood, EL III. Alternatives to clam shell as cultch for oysters and the use of gypsum for the production of cultchless oysters. *Journal of Shellfish Research*. 1991; 10:405–410.

53. La Peyre, M, Furlong, J, Brown, LA, Piazza, BP, Brown, K. Oyster reef restoration in the northern Gulf of Mexico: Extent, methods and outcomes. *Ocean and Coastal Management.* 2014; 89: 20–28.

Figure captions

Figure 1. Total number of juvenile oysters consumed across MPE treatments for limestone and shell substrates. MPE treatments: Control = no predators present; SC = Stone crab; TF/SC = toadfish and stone crab; MC = mud crab; SC/MC = stone crab and mud crab; TF/MC = toadfish and mud crab; TF/SC/MC = toadfish, stone crab and mud crab.

Figure 2. TMII experiment. Consumption of Juvenile oysters by three size classes of mud crabs across three caged predator treatments, empty, toadfish and stone crab.

Ethics Statement

This study was carried out in strict accordance with the recommendations in the Guide for the Care and Use of Laboratory Animals of the National Institutes of Health. The protocol for this study was approved by the Institutional Animal Care and Use Committee (IUCUC) of the University of Southern Mississippi (Protocol Number: 16052606), based on an Animal Subjects Research Application submitted to the USM Office of Research Integrity. In addition, VRF completed the Collaborative Institutional Training Initiative Program (CITI Program). Certificates were obtained for both the animal subjects course and the common course covering various considerations related to ethical conduct in all professional and research activities. All efforts to obtain, handle and house fish and invertebrate subjects were made humanely. Following the completion of experiments, fish subjects were released back into the wild. Field work was not done in protected or privately owned areas, and no protected species were sampled. Collections of reef animals were made under the auspices of a Scientific Research Permit issued to the USM Gulf Coast Research Laboratory by the Mississippi Department of Marine Resources (MSDMR).

Data Availability Statement

Data used for this study were obtained from experiments as described in the Methods and materials section of this article. All data were collected, compiled, curated and validated by V.R. Fleer. If accepted for publication, data used for this study, including metadata formats, will be submitted as excel files exported from SPSS 25.0 files to the GRIIDC data archive, at

https://data.gulfresearchinitiative.org/, hosted by the Harte Research Institute for Gulf of Mexico Studies of Texas A&M University-Corpus Christi and The Gulf of Mexico Research Initiative (GoMRI).

Funding

Research for this article was conducted as part of a Ph.D. Dissertation by VRF, and was not supported by any specific grant from funding agencies in the public, commercial, or not-for-profit sectors. Supplies for this research project were largely provided to VRF by the Mississippi Department of Marine Resources.

Competing interests

The authors have no competing interests.

Click here to access/download;Figure;PlosFigure1.tif

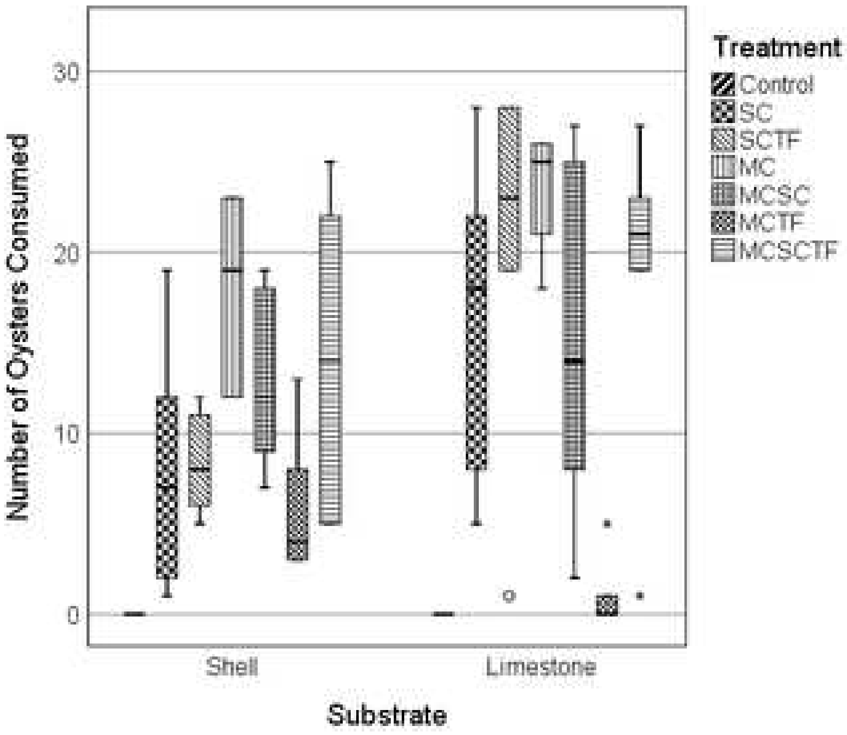

Figure 2 - Fleer and Rakocinski

Click here to access/download;Figure;PlosFigure2.tif

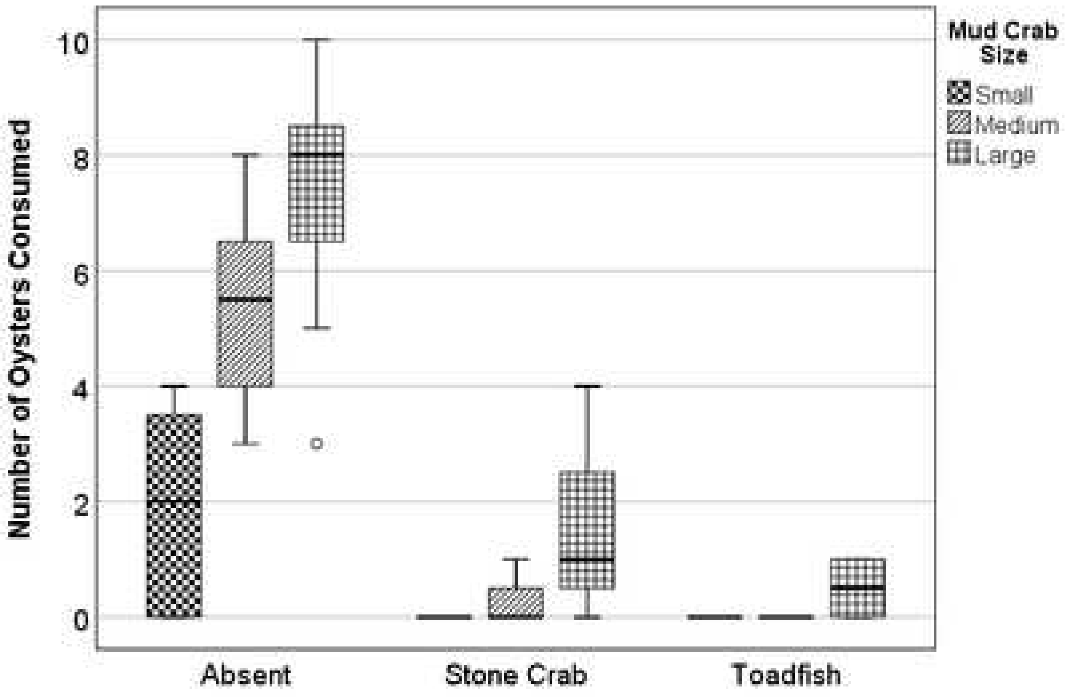

Supplement: Attachment — Submitted filename: PONE-D-20-05873_reviewer (1).pdf [file pone.0242965.s002.pdf]
