# Supplementary material for: Destabilizing effects on a classic tri-trophic oyster-reef cascade
Source: PLoS One. 2020 Dec 15;15(12):e0242965. doi: 10.1371/journal.pone.0242965 (PMC7737904; doi:10.1371/journal.pone.0242965)
Supplement: Attachment — Submitted filename: Editor comments on Revised Manuscript with Track Changes.docx [file pone.0242965.s003.docx]

***Destabilizing effects on a classic tri-trophic oyster-reef cascade***

Virginia R. Schweiss, Chet F. Rakocinski*

Division of Coastal Sciences, School of Ocean Science and Engineering, University of Southern Mississippi, Gulf Coast Research Laboratory, Ocean Springs, MS, United States of America

*Corresponding author:

Email: [chet.rakocinski@usm.edu](mailto:chet.rakocinski@usm.edu)

Short Title: Destabilizing effects on a trophic cascade.

**Abstract**

How interactions among multiple predators affect the stability of trophic cascades is a topic of special ecological interest. To examine factors affecting stability of the classic tri-trophic oyster reef cascade within a novel context, configurations of three predators, including the Gulf toadfish, Gulf stone crab, and oystershell mud crab, were manipulated together with oyster shell or limestone gravel substrate within a multiple predator effects (MPE) experiment. In addition, separate trait-mediated-indirect interaction (TMII) experiments examined the degree of inhibition of oyster consumption with respect to mud-crab size and top predator identity in the absence of multiple cues and extraneous factors. The classic tri-trophic cascade formed by the toadfish-mud crab-oyster configuration was potentially weakened by several interactions within the MPE experiment. Consumption of oysters and mud crabs by the intraguild stone crab was undeterred by the presence of toadfish. Although mud crab feeding was inhibited in the presence of both toadfish and stone crabs, estimated non-consumptive effects (NCEs) were weaker for stone crabs in the MPE experiment. Consequently, the total effect was destabilizing when all three predator species were together. Weaker inhibition of mud crab feeding was inversely related to higher direct predation on mud crabs within the MPE experiment. Non-parallel TMII experiments revealed exacerbated inhibition of mud crab feeding in response to stone crabs under sparse conditions. TMII experiments also implied that inhibition of mud crab feeding could largely account for NCEs within multiple predator settings, as opposed to interference involving other mud crabs or top predators. An inverse relationship between mud crab size and NCE strength in the TMII experiment disclosed an additional potentially destabilizing influence on the tri-trophic-cascade. Finally, although habitat complexity largely dampened the consumption of oysters across MPE treatments, complex habitat reduced the inhibition of mud crab feeding in the presence of toadfish. This study underscores how some ecological interactions can mediate trophic cascades and provides additional insights about the trophic dynamics of oyster reefs for further testing under natural conditions.

**Introduction**

Communities are structured through strong biotic interactions, including top-down effects of predation [1,2]. Top predators may stabilize basal prey populations (i.e., decrease chances of basal prey extirpation) by suppressing the predation interaction between intermediate predators and basal prey within simple tri-trophic cascades [3,4,5,6]. However, the stability of trophic cascades can become less predictable as more interacting species are added [7]. How the stability of trophic cascades is affected by interactions among multiple predator species is a topic of special ecological interest [8,9].

Top predators indirectly enhance abundances of basal prey within trophic cascades [10]. Basal prey benefit indirectly from top predators through their consumption of intermediate predators, known as density-mediated indirect interactions (DMIIs) [11,12,13,14], as well as through suppression of feeding by intermediate predators, known as trait-mediated indirect interactions (TMIIs) [3,5,6]. Non-consumptive effects (NCEs) arising from consequent non-lethal changes in the biology or behavior of intermediate predators lead to their reduced fitness [13,15,16]. For example, reduced feeding due to predator avoidance will allow less energy for growth or reproduction [14,17].

Multiple Predator Effects (MPEs) arise from facilitative and competitive interactions among multiple predators that engender non-additive effects on basal prey [18,19]. Synergistic MPEs cannot be predicted from effects of single predators in isolation [14,20]. Moreover, interactions among multiple predators may affect the stability of trophic cascades through basal prey mortality [21]. Facilitation between top predators can destabilize trophic cascades by accelerating feeding on a basal resource [19]. Conversely, interference or predation between intraguild predators (i.e., species feeding on a common prey) [14,22] or size classes can decelerate feeding on a common basal resource [23]. Thus, to discern the combined effects on shared basal prey, multiple predators need to be considered jointly [9].

Trophic cascade stability responds to any traits that mediate the risk of mortality to intermediate predators [24]. Body size is one overarching trait often overlooked within food webs [25]. Inhibitive effects of top predators on feeding by intermediate predators should vary with body size, because predation risk correlates directly with the predator-prey size ratio [22]. As such, TMII strength should vary with the size of intermediate predators [26]. Accordingly, predator interactions may induce changes in the size selection of basal prey [20]. Additionally, interference or predatory interactions among intraguild predators are strongly mediated by body size, which adds a horizontal dimension within food webs [23].

Habitat complexity also mediates trophic cascades by weakening or reinforcing predation and interference interactions [14,27,28,29]. Structurally complex habitat reduces predator efficiency by providing refuge for basal prey as well as for intermediate predators [30,31,32,33,34]. Structurally complex habitat often stabilizes trophic cascades by reducing predation on basal prey [17,35,36]. Conversely, interference may be mitigated within complex habitat by reducing the frequency of encounters between intraguild predators [22,29,36,37,38]. Given that, the degree to which the stability of trophic cascades is weakened or strengthened by habitat complexity also depends on the identities and body sizes of intermediate and top predators [9].

The oyster reef complex offers an ideal model system for the study of trophic interactions in connection with basal oyster prey [9,14,19,22,23]. Core assemblages comprising multiple species and sizes of top and intermediate predators can be readily manipulated [35]. Interference, predation and facilitation within assemblages can modulate abundances of basal oysters in surprising ways [19]. Trophic interactions are also mediated by habitat complexity [14]. Previous studies of oyster reef trophic cascades reveal important context dependence with respect to predator identity [9,19], predator size [22,23] and habitat complexity [14,22].

Experimental studies of oyster reefs typically examine subsets of predators forming tri-trophic cascades [sensu 17] or triangular food web compartments [sensu 19], within which effects of competitive, predatory, or facilitative interactions influence cascade stability [39]. A classic tri-trophic cascade within the oyster-reef complex comprises the oyster toadfish (*Opsanus tau*), the mud crab (*Panopeus* spp.), and the juvenile oyster (*Crassostrea virginica*). Oyster toadfish can stabilize the trophic cascade by inhibiting mud crab feeding on juvenile oysters, although the efficacy of the cascade can be context dependent in the field [40]. How other predator species interact can also affect the stability of the tri-trophic cascade [14].

A common experimental approach to disentangling MPEs involves manipulating combinations of different types and sizes of predators [22,23]. As such, outcomes of complex interactions have not been examined for the multiple predator configuration involving the toadfish, the stone crab, and the mud crab. As top predators, toadfish consume mud crabs and juvenile stone crabs, but not oysters [41]. Stone crabs are intraguild predators [sensu 42] of mud crabs [9], and mud crabs potentially interfere with [14] and consume each other [23]. Both mud crabs and stone crabs share juvenile oysters as prey [43].

Previous studies illustrate how various ecological interactions can affect the stability of the oyster reef tri-trophic cascade [9, 17, 19, 22, 23]. Here we focus on several effects on the oyster tri-trophic cascade in a novel context, including addition of the intraguild predator, Gulf stone crab (*Menippe adina*), habitat complexity characterized by oyster shell vs. limestone gravel substrate and TMII effects of toadfish vs. stone crab relative to mud crab size. To examine these effects, two unrelated sets of laboratory experiments were conducted. Effects of predator identity configuration and habitat complexity were examined through mesocosm trials comprising exclusive combinations of Gulf toadfish (*Opsanus beta*), Gulf stone crab (*Menippe adina*), the oystershell mud crab (*Panopeus simpsoni*), two size classes of juvenile oysters, and two substrate types (oyster shell vs. limestone gravel). Trait mediated indirect interaction (TMII) effects attributable strictly to inhibition of mud crab feeding by top predators were examined through experiments involving caged toadfish or stone crabs as top predators and different sizes of mud crabs as intermediate predators.

**Materials and methods**

**Sources of test animals**

Animals for experiments were obtained using plastic sampling trays (52 cm x 52 cm x 11 cm) lined with self-closing mosquito netting (0.5 mm mesh) and filled with oyster shell (≈ 6,000 g). Trays were deployed *in situ* for 10 weeks at twelve nearshore subtidal reefs from located along the entire Mississippi coast [44]. Sites were collocated as pairs of historically harvested and low-profile substrate-augmented reefs geographically dispersed within the western, central, and eastern regions of the Mississippi coast, falling between just west of Saint Louis Bay (30.30030° N, -89.32930° W) and near the mouth of the Pascagoula River (30.36453° N, -88.59988° W).

Live animals for experiments were identified, measured, and placed into holding tanks. Test animals were acclimated at 25°C and 29 salinity for at least three days. Individual crabs were kept isolated to prevent aggressive interactions. Reared juvenile oysters for experiments were obtained from the University of Auburn Shellfish Laboratory in Dauphin Island, AL.

**MPE experiment**

Oyster survival was examined in response to different combinations of top and intermediate predators, and two substrate types, oyster shell or #57 limestone gravel, which are commonly used for oyster reef restoration. Substrate was soaked and cleaned to remove organic debris before use. Small mesocosms (89 x 48 x 33 cm tubs) filled with 110 l filtered seawater served as experimental units. One hour prior to the introduction of test animals, 30 loose juvenile oysters (15 spat, 15 mm shell height (SH) and 15 seed, 30 mm SH) were randomly distributed on the substrate. Heaters maintained the water temperature at 25° C; aerators provided water circulation and oxygenation. Abiotic variables were monitored with a YSI model 85 handheld meter. Sizes of test animals were standardized: mud crabs ≈ 23 mm carapace width (CW), stone crabs ≈ 60 mm CW, and toadfish ≈ 120 mm total length. Top predators matched the most common sizes sampled from source reefs and capable of consuming mud crabs. Oyster prey densities ensured total prey depletion did not occur within the 24 h trial duration. Four randomized blocks comprising all MPE treatment combinations were run separately for each substrate type: (1) 30 oyster (control); (2) seven mud crab (MC); (3) seven MC and one toadfish (TF); (4) seven MC and one stone crab (SC); (5) one SC; (6) one SC and one TF; (7) seven MC, one TF, and one SC. The TF and oyster MPE treatment was not included, as toadfish do not consume oysters [40, 41]. After trials, surviving oysters and mud crabs were enumerated. Test animals were not reused to ensure independence.

**TMII experiments**

Non-consumptive effects (NCE) estimated from the MPE experiment (see below) could result from multiple causes including inhibition or interference behavior and interactions between top and intermediate predators, as mediated by substrate complexity. Thus, an unrelated complementary set of trait-mediated indirect interaction (TMII) experiments focused on responses by different sizes of intermediate mud crabs under sparse conditions. Tanks (76 x 33 x 33 cm) filled with 75 l of filtered seawater at 29 salinity served as experimental units. Heaters regulated the water temperature at 25° C and overhead LED lights were kept on a 12-h light cycle. To isolate TMII effects, toadfish or stone crabs were retained within cylindrical (18 cm diameter x 30.5 cm tall) 2.6 cm mesh wire cages. Ten loose 15 mm SH oysters were distributed within each unit prior to the introduction of individual mud crabs belonging to one of three size classes: small (13-15 mm CW), medium (21-23 mm CW), or large (28-31 mm CW). The use of individual mud crabs precluded intraspecific interference and cues. Empty cage treatments controlled for cage effects. Substrate was withheld to strictly isolate responses to the presence of caged predators. Each of four randomized blocks was comprised of three experimental units containing individual mud crabs of each size class in the presence of a caged predator (i.e., stone crab ≈ 65 mm carapace width; toadfish ≈ 120 mm TL ), in addition to three control units containing individual mud crabs of each size class and empty cages. The duration of each run was 24 h, after which surviving oysters were enumerated. All runs for each type of top predator ensued over consecutive 4-d periods.

A second TMII experiment focused on interactions involving one large (28-30 mm CW) and three small (≈ 14-15 mm CW) mud crab predators, exposed together to either caged toadfish (120 mm TL) or empty control cages. Experimental conditions were identical to those for individual mud crab TMII experiments. Runs ensued for 24 h, with replacement of consumed oysters after 12 h due to high feeding rates. Surviving oysters were enumerated at 12 and 24 h.

**Ethics Statement**

This study was carried out in strict accordance with the recommendations in the Guide for the Care and Use of Laboratory Animals of the National Institutes of Health. The protocol for this study was approved by the Institutional Animal Care and Use Committee (IUCUC) of the University of Southern Mississippi (Protocol Number: 16052606), based on an Animal Subjects Research Application submitted to the USM Office of Research Integrity. In addition, VRS completed the Collaborative Institutional Training Initiative Program (CITI Program). Certificates were obtained for both the animal subjects course and the common course covering various considerations related to ethical conduct in all professional and research activities. All efforts to obtain, handle and house fish and invertebrate subjects were made humanely. Following the completion of experiments, fish subjects were released back into the wild. Field work was not done in protected or privately-owned areas, and no protected species were sampled. Collections of oyster reef animals were made under the auspices of a Scientific Research Permit issued to the USM Gulf Coast Research Laboratory by the Mississippi Department of Marine Resources (MSDMR).

**Statistical analysis**

For MPE experiments, the mean numbers of consumed oysters and consumed mud crabs were dependent variables within separate two-way ANOVAs with Treatment and Substrate as crossed fixed factors using the General Linear Models procedure in SPSS (version 25). Both small and large oysters were pooled as a single response, as survival of both size classes was strongly positively correlated (r = 0.825; P_1t_ < 0.001) and differences in numbers of large vs. small oysters consumed were lacking among the seven MPE treatment levels. Numbers of small and large oysters consumed were not different across any of the MPE treatments (paired t-tests; Holm-Bonferroni sequential corrected rejection at α < 0.05 [45]). The MPE treatment control (i.e., oyster alone) was excluded from the GLM ANOVA to avoid an omnibus test that was driven by the lack of observed oyster mortality for that treatment. Levene’s and F-tests for equality of error variances and heteroscedasticity were not different for oyster mortality within the MPE ANOVA. Follow-up LSD tests among factor levels were interpreted based on Holm-Bonferroni sequential adjusted rejection levels [45]. The ANOVA for the mean number of mud crabs consumed involved the four MPE treatment levels with mud crabs. Because error variance in the response was heterogeneous (Levene Statistic = 6.465; P < 0.001) the mud crab mortality response was tested relative to treatment and substrate with a two-way PERMANOVA using Manly’s approach—Unrestricted Permutation of Observations in R.

The ANOVA in the first TMII experiment examined the mean number of oysters consumed across the three mud crab size levels (i.e., small, medium, large) crossed with three predator levels (i.e., stone crab (n=12), toadfish (n=12), no predator (n = 24)). Because error variance in the response was heterogeneous (Levene Statistic = 4.925; P < 0.001), oyster consumption was examined relative to mud crab size and predator factors and their interaction with a two-way PERMANOVA using Manly’s approach. For the second TMII experiment involving the mixed-size mud crab treatment, an independent two-sample t-test compared numbers of oysters consumed between the two groups (toadfish present vs. toadfish absent). Variance in the response was not heterogeneous between the groups (F = 0.866; P = 0.388).

Risk reduction or enhancement with respect to oyster and mud crab survival was examined for various treatments from the MPE experiment with one-sample t-tests, using a multiplicative model approach defined by Soluk and Collins [15] and Sih et al. [18]. Expected oyster mortality values were adjusted for numbers of predated mud crabs (i.e., 1.464 oysters per mud crab based on ½ the number of mud crabs lost), to adjust for oysters that would have been consumed by consumed mud crabs [14]. No such adjustment was needed for mud crab as prey, as mud crab cannibalism did not occur. Toadfish predation on oysters was assumed zero for calculations of expected numbers of oysters consumed. One-tailed t-tests reflected directionality in the expected outcomes, as indicated by the relative difference between expected and observed values. For the MPE combination involving all three predators (i.e., TF-SC-MC) for which oysters were prey, two different one-sample t-tests were based on expected values generated using either the three single predator outcomes or the two top predator and mud crab combinations. In addition, for the MPE combination involving all three predators (i.e., TF-SC-MC) for which mud crabs were prey, two different one-sample t-tests were based on expected values generated using single stone crab and toadfish outcomes from both substrates pooled as well as from only limestone substrate, as there was a strong interaction with substrate. Where risk reduction was inferred by observed values that were less than expected values, consumptive effect (CE) and non-consumptive effect (NCE) proportions were calculated [14].

For the TMII experiments, non-consumptive effect (NCE) and consumptive effect (CE) proportions were obtained directly from the difference in oyster mortality between caged predator and empty cage treatments. The design of TMII experiments ensured that NCEs were attributable exclusively to the presence of top predators. However, due to incommensurate designs, the MPE and TMII experiments were not directly comparable.

**Results**

**MPE Experiment**

The total number of oysters consumed differed for both main factors, but not for the interaction between MPE Treatment and Substrate (Table 1). Overall, more oysters were consumed in limestone (15.6 ± 1.93 se) than in shell (11.3 ± 1.27 se). And more oysters were generally consumed in limestone across MPE treatments, except for the toadfish/mud crab (TF/MC) treatment for which five-fold more oysters were consumed in shell than in limestone (t = 3.14; P_1-t_ = 0.007) (Table 2; Figure 1). The largest between-treatment difference in the mean number of oysters consumed (i.e., 16.8) pooled across both substrates exemplified the strong effect of toadfish on the consumption of oysters by mud crabs (Table 2). The lowest average number of oysters consumed for the TF/MC treatment (i.e., mean ± se = 3.7 ± 1.32) was less than that for the MC (Holm-Bonferroni sequential adjusted LSD tests, P < 0.001), TF/SC (P = 0.003), SC/MC (P = 0.003) and TF/SC/MC (P = 0.001) treatments (Table 2). Comparable numbers of oysters were consumed across all treatments which involved stone crabs as one of the predators (mean ± se = 12.2 ± 2.89 SC; 14.1 ± 2.56 MC/SC; 14.1 ± 3.09 SC/TF). Thus, stone crab predation on oysters was not inhibited by the presence of toadfish. Moreover, the combined effect of mud crabs and stone crabs on oyster mortality was not additive (mean ± se = 20.5 ± 1.65 MC; 14.1 ± 2.56 MC/SC), likely pointing to some effects of inhibition and predation of mud crabs by stone crabs. Nominally more oysters were consumed by mud crabs when alone than by stone crabs when alone (mean ± se = 20.5 ± 1.65 for 7 MC; 12.2 ± 2.89 for 1 SC).

**Table 1. Two-way ANOVA of the total number of oysters consumed within the MPE experiment (bold P ≤ 0.05).**

| **Source** | **Type III Sum of Squares** | **df** | **Mean Square** | **F** | **P** | **Partial Eta Squared** | **Observed Power^b^** |
| --- | --- | --- | --- | --- | --- | --- | --- |
| **Corrected Model** | 2219.73 | 11 | 201.79 | 3.56 | **0.001** | 0.45 | .99 |
| **Intercept** | 10881.07 | 1 | 10881.07 | 191.79 | **< 0.001** | 0.80 | 1.00 |
| **Substrate** | 281.67 | 1 | 281.67 | 4.97 | **0.031** | 0.09 | 0.59 |
| **MPE Treatment** | 1547.33 | 5 | 309.47 | 5.46 | **< 0.001** | 0.36 | 0.98 |
| **Substrate × Treatment** | 390.73 | 5 | 78.15 | 1.38 | 0.249 | 0.13 | 0.44 |
| **Error** | 2723.20 | 48 | 56.73 |  |  |  |  |
| **Total** | 15824.00 | 60 |  |  |  |  |  |
| **Corrected Total** | 4942.93 | 59 |  |  |  |  |  |

**Table 2. Number of oysters consumed (Mean ± SD) for MPE treatment levels for oyster shell and limestone gravel substrata, as well as for totals across treatments and substrata (maximum of 30 possible).**

| **Substrate** | **MPE Treat** | **Mean** | **Std. Deviation** |
| --- | --- | --- | --- |
| **Shell** | **SC** | 8.20 | 7.46 |
|  | **TF/SC** | 8.40 | 3.05 |
|  | **MC** | 17.80 | 5.54 |
|  | **SC/MC** | 13.00 | 5.34 |
|  | **TF/MC** | 6.20 | 4.32 |
|  | **TF/SC/MC** | 14.20 | 9.31 |
|  | **Total** | 11.30 | 6.96 |
| **Limestone** | **SC** | 16.20 | 9.60 |
|  | **TF/SC** | 19.80 | 11.17 |
|  | **MC** | 23.20 | 3.56 |
|  | **SC/MC** | 15.20 | 10.76 |
|  | **TF/MC** | 1.20 | 2.17 |
|  | **TF/SC/MC** | 18.20 | 10.06 |
|  | **Total** | 15.63 | 10.59 |
| **Total** | **SC** | 12.20 | 9.14 |
|  | **TF/SC** | 14.10 | 9.78 |
|  | **MC** | 20.50 | 5.23 |
|  | **SC/MC** | 14.10 | 8.09 |
|  | **TF/MC** | 3.70 | 4.17 |
|  | **TF/SC/MC** | 16.20 | 9.38 |
|  | **Total** | 13.47 | 9.15 |

MPE Treat = Multiple Predator Effect treatments.

SC = stone crab.

TF/SC = toadfish and stone crab.

MC = mud crab.

SC/MC = stone crab and mud crab.

TF/MC = toadfish and mud crab.

TF/SC/MC = toadfish, stone crab and mud crab.

**Table 3. MPE treatment risk-model t-tests.**

| **MPE Treat** | **Prey type** | **Exp N** | **MC N** | **Obsv N** | **SE Obs** | **t** | **P_1-t_** | **CE** | **NCE** |
| --- | --- | --- | --- | --- | --- | --- | --- | --- | --- |
| TF+MC | Oyster | 19.04 | 20.50 | 3.70 | 1.32 | -11.645 | **<0.001** | 0.19 | 0.81 |
| SC+MC | Oyster | 24.36 | 22.46 | 14.10 | 2.56 | -3.268 | **0.005** | 0.63 | 0.37 |
| TF+SC | Oyster | 12.20 | NA | 14.10 | 3.10 | 0.614 | 0.277 | NA | NA |
| TF+SC+MC_a_ | Oyster | 24.36 | 21.58 | 16.20 | 2.97 | -1.814 | 0.051 | 0.75 | 0.25 |
| TF+SC+MC_b_ | Oyster | 16.06 | 13.28 | 16.20 | 2.97 | 0.985 | 0.175 | NA | NA |
| TF+SC+MC_c_ | Mud crab | 3.23 | NA | 3.80 | 0.58 | 0.980 | 0.191 | NA | NA |
| TF+SC+MC_d_ | Mud crab | 2.11 | NA | 1.90 | 0.69 | -0.310 | 0.381 | 0.90 | 0.10 |

MPE Treat = Multiple Predator Effect treatment; Exp N = expected number of prey; MC N = expected number of prey, corrected for lost mud crabs; Obsv N = observed number of prey; SE Obs = standard error of observed number prey; CE = consumptive effect; NCE = non-consumptive effect.

Negative t-values connote MPE risk reduction for basal prey; Positive t-values imply MPE risk enhancement for basal prey; 1-tailed t-tests imply expected directionality in the outcomes; bold P ≤ 0.05.

Correction for Mud Crab (MC N) = 1.464 oysters lost per individual mud crab.

NCE not applicable for mud crabs as prey because mud crab cannibalism was not observed.

Toadfish predation on oysters assumed zero.

^a^TF+SC+MC_a_ – toadfish, stone crab and mud crab MPE relative to three single predator combinations.

^b^TF+SC+MC_b_ – toadfish, stone crab and mud crab MPE relative to two top predators together plus mud crab combination.

^c^TF+SC+MC_c_ – toadfish, stone crab and mud crab MPE from limestone treatments.

^d^TF+SC+MC_d_ – toadfish, stone crab and mud crab MPE from both substrates.

CE and NCE are not applicable (NA) when Obs N > Exp N (i.e., positive t value).

The number of mud crabs consumed within the MPE experiment strongly differed for both main factors as well as for the interaction between MPE Treatment (i.e., the four MPE treatments with mud crabs) and Substrate (two-way PERMANOVA, MPE P = 0.0026; Substrate < 0.001; MPE × Substrate = 0.001). Except for the TF/MC treatment, mud crab loss did not occur within shell substrate, nor for the MC treatment within limestone substrate. Thus, mud crab cannibalism did not occur during the 24 h experimental period. Mud crab loss did occur for almost every treatment involving toadfish, except for the treatment comprising both top predators in shell substrate.

The interaction effect reflected uneven and higher loss of mud crabs in limestone gravel than in oyster shell for the MPE treatments involving stone crabs (i.e., SC/MC and TF/SC/MC). Mud crab loss in limestone averaged more than two-fold higher when the stone crab was the only top predator than when toadfish was the only top predator (mean ± se= 2.6 ± 1.03 vs. 1.0 ± 0.45). And mud crab loss was comparable across both substrate types when toadfish was the only top predator (mean ± se = 1.0 ± 0.45 for both substrates). Accordingly, mud crab feeding was more inhibited by toadfish when it was the only top predator (oyster NCE = 0.81) compared to when the stone crab was the only top predator (oyster NCE = 0.37). And mud crab feeding was least inhibited when both top predators were present (oyster NCE = 0.25) (Table 3). As such, the greatest loss of mud crabs (mean ± se = 3.80 ± 0.58) occurred when both top predators were present in limestone gravel. And mud crab loss was essentially additive relative to the two top predators, whether considering just limestone (exp N = 3.23 vs. obs N = 3.80) or both substrates pooled (exp N = 2.11 vs. obs N = 1.90) (Table 3). The lack of much difference between observed and expected loss of mud crabs in the presence of both top predators as opposed to individually for limestone and both substrates pooled resulted in weak NCEs (Table 3).

**TMII experiments**

For the TMII experiment on mud crab size, the number of oysters consumed strongly differed for both main factors as well as for the interaction between mud crab size and predator (two-way PERMANOVA, Predator P < 0.001; MC Size P < 0.001; Predator × MC Size = 0.003). The interaction effect reflected non-parallel declines in numbers of oysters consumed with crab size across the three predator levels (Fig. 2). Markedly more oysters were consumed with increasing crab size within the empty cage treatment, whereas oyster consumption was greatly inhibited across the three crab sizes within the caged stone crab and toadfish treatments. Oyster consumption was roughly an order of magnitude higher in the absence of top predators and substrate: the mean number of oysters consumed across all size classes of mud crabs was 4.87 ± 0.31 se in empty cages vs. 0.58 ± 0.44 se and 0.17 ± 0.44 se in the presence of stone crab and toadfish, respectively. Oyster consumption by mud crabs appeared somewhat more inhibited by toadfish than by stone crabs (Fig. 2). For the TMII experiment using mixed sizes of mud crabs, almost four-fold fewer oysters were consumed in the presence of caged toadfish (4.5 ± 2.1 se) than when cages were empty (16.8 ± 3.3 se) (t = 3.165; P_1t_ = 0.009).

Non-consumptive effects (NCE) due to inhibition of oyster consumption varied with mud crab size, and were less pronounced for stone crab than for toadfish (Table 4). A discernable trend in NCE strength with mud crab size was apparent when data were pooled for both top predators (Table 4). Large mud crabs showed the lowest NCE values, although NCEs were relatively high for all three size classes (i.e., all > 0.79). The NCE value for the mixed-size TMII experiment was lower than that for the large mud crab treatment from the first TMII experiment in the presence of toadfish (NCE = 0.73 vs. 0.93), implying interference among mud crabs did not noticeably inhibit the consumption of oysters even in the absence of substrate.

**Table 4. Consumptive effects (CE) vs. non-consumptive effects (NCE = 1 – CE) for caged predator TMII experiments.**

| **Treatment** | **Predator**  **Present** | **Predator**  **Absent** | **CE** | **NCE** |
| --- | --- | --- | --- | --- |
| SC + MC_L_ | 1.50 | 7.375 | 0.203 | 0.797 |
| SC + MC_M_ | 0.25 | 5.375 | 0.046 | 0.953 |
| SC + MC_S_ | 0.00 | 1.875 | 0.000 | 1.000 |
| TF + MC_L_ | 0.50 | 7.375 | 0.068 | 0.932 |
| TF + MC_M_ | 0.00 | 5.375 | 0.000 | 1.000 |
| TF + MC_S_ | 0.00 | 1.875 | 0.000 | 1.000 |
| BOTH+MC_L_ | 1.00 | 7.375 | 0.136 | 0.864 |
| BOTH+MC_M_ | 0.125 | 5.375 | 0.023 | 0.977 |
| BOTH+MC_S_ | 0.00 | 1.875 | 0.000 | 1.000 |
| TF + MC_L/S_ | 4.50 | 16.75 | 0.269 | 0.731 |

BOTH = data pooled for caged toadfish and stone crab.

L, M, and S = large, medium, and small mud crab.

L/S = mixed size mud crab TMII experiment.

**Discussion**

Studies of MPEs within oyster reef assemblages typically focus on how interspecific interactions affect the stability of the oyster reef tri-trophic cascade through non-additive predator effects [15]. In addition to predator identity [9], other ecological factors that mediate the stability of the oyster-reef trophic cascade include body size [23], predator density [46], and habitat complexity or patchiness [14, 47]. As such, the stability of a given oyster-reef configuration is evaluated within the context of unique combinations of ecological factors [9,36]. Even given its tractable nature, considering the multitude of trophic interactions within the oyster-reef food web would be unwieldy. So, experimental factors are selected prudently to obtain new insights into oyster-reef trophic stability. Here, we chose to examine the tri-trophic toadfish-mud crab-oyster cascade in connection with the interactive role played by the stone crab, an intraguild predator and common resident of Gulf of Mexico (GoM) oyster reefs.

As often shown by other studies, the toadfish was a primary stabilizing influence on the tri-trophic cascade in our study. However, unlike Grabowski et al. [14], where the tri-trophic cascade was reinforced by interactions between toadfish and the generalist blue crab capable of consuming both intermediate predators and basal oyster prey, the addition of the intraguild stone crab weakened the tri-trophic cascade in our study. Toadfish did not inhibit stone crab feeding in our study. Generalist predators have destabilized trophic cascades in other oyster reef studies [48]. In addition to indirectly suppressing feeding by mud crabs on oysters through both consumption of mud crabs and inhibition of mud crab feeding behavior, stone crabs directly consumed oysters as a shared prey. However, mud crab feeding behavior was less strongly inhibited by stone crabs than by toadfish in the MPE experiment. Further weakening of the trophic cascade was indicated by lower inhibition of mud crab feeding when all three predators were together (NCE=0.25) than when toadfish (NCE=0.81) or stone crabs (NCE=0.37) occurred alone with mud crabs. Moreover, the cascade was not stabilized by intraspecific interference and cannibalism among mud crabs, as in Geraldi [23]. Thus, the trophic balance was generally destabilized when all three predators were together, as compared to when toadfish and mud crabs were the only two predators.

Toadfish stabilized the trophic cascade mainly by inhibiting mud crab feeding on oysters rather than by directly consuming mud crabs. Indeed, direct predation on mud crabs was higher for stone crab than for toadfish in limestone gravel. But oyster consumption by mud crabs was not inhibited as much by stone crabs as by toadfish, as shown by a more than two-fold difference in NCEs within the MPE experiment. Nevertheless, the combined effect of predation and inhibition of mud crabs by stone crabs carried over to a nonadditive effect of fewer oysters consumed than expected when both intraguild crabs were together. In contrast, consumptive effects of toadfish and stone crabs on mud crabs were additive when all three predators were together. Previous studies provide some insights. Toadfish can induce mud crabs to seek refuge and limit their movements [14]. Stone crabs are more adept at flushing mud crabs from refuges, whereas toadfish are ambush predators [47]. Consequently, predation on mud crab was highest in the presence of both top predators. The disposition of stone crabs as a stabilizing influence on the trophic balance may depend on the profitability of oysters and mud crabs as prey. Depending on the costs and benefits as a function of predator-prey size vs. handling time, stone crabs may prefer mud crabs or oysters as more profitable prey types, but this needs to be tested. Both mud crabs and oysters were consumed by stone crabs in our study.

It was expected that toadfish might inhibit stone crab feeding behavior, because as common residents of GoM oyster reefs juvenile stone crabs regularly occur in toadfish diets [41]. However, toadfish did not inhibit the consumption of mud crabs by stone crabs in our MPE experiment. Nor did toadfish inhibit the consumption of oysters by stone crabs in the absence of mud crabs. The sizes of stone crabs used in our MPE experiment matched the most common size class that could consume adult mud crabs. Even though the total lengths of MPE toadfish (i.e., 120 mm TL) were about two-fold greater than the carapace widths of MPE stone crabs, MPE stone crabs may have been too large to perceive much risk of toadfish predation. However, toadfish can grow large enough (i.e., > 300 mm TL) [23] to pose a predatory risk to adult stone crabs in the field, implying stone crabs of the size used in our experiment would not have ontogenetically outgrown the risk of predation by toadfish.

Body size is an important ecological factor mediating the stability of food webs through predator-prey interactions between top and intermediate predators [49] and between intermediate or intraguild predators and basal prey [20,43,48]. Body size also mediates interference competition or predation among intraguild predators [22,23]. For example, size-selection of hard clams (*Mercenaria*) proved important for understanding the preference of small clams by blue crabs, but was not important for stone crabs with stronger crushing ability. Notwithstanding the disparate body sizes of intermediate mud crabs and intraguild stone crabs, size selection of oysters was not apparent for either species of crab in our MPE experiment. The ratio of small (i.e., 15 mm spat) to large (i.e., 30 mm seed) oysters consumed for stone crabs vs. mud crabs when alone was 45%:55% and 55%:45%, respectively. Additionally, numbers of small vs. large oysters consumed did not differ across MPE treatments. But when considered over a wide range of sizes (i.e., up to 70 mm VLV), costs associated with handling time or crushing success of hard shell molluscan prey increases with predator-prey size ratio, and as such can affect the feeding preferences of stone crabs, blue crabs, and mud crabs [43]. A size threshold below 25 mm SH serves as a refuge from stone crab predation [43]. However, small oysters below 25 mm SH were vulnerable to predation by mud crabs. In that study, larger stone crabs (80-130 mm CW) than those in our MPE experiment (60 mm CW) were used. And mud crabs (23 mm CW) readily consumed oysters in both size classes in our MPE experiment. Indeed, both stone crabs and mud crabs possess strong crushing chelae. However, the use of loose oysters rather than oysters attached within a reef matrix limits the applicability of our experiment.

Risk perception by intermediate predators is also mediated by top predator body size [22] and top predator biomass where aqueous chemical cues are concerned [54]. Accordingly, size-related NCE release was detected for large mud crabs in the TMII experiment, as indicated by relatively higher oyster consumption in the presence of top predators. Plasticity in the expression of TMIIs presents an important influence on trophic cascades [13]. Different sizes of intermediate predators should respond commensurately with perceived threat of predation, as a balance between risk and energetic trade-offs [6]. Thus, TMII strength can vary with the intermediate predator size [26], and the size structure of the intermediate predator population can mediate predation pressure on prey populations. The finding of size-related NCE release for mud crabs complements Geraldi [23], who showed that the oyster-reef trophic cascade was mediated by the size composition and density of intermediate mud crabs. But in contrast to our study, Geraldi noted that the trophic cascade was stabilized by interference and cannibalism between mud crabs. Surprisingly, mud crab interactions exerted as much influence on trophic stability as did inhibition of mud crab feeding by the presence of a top predator [23]. Neither interference nor cannibalism occurred among mud crabs in our study. However, Geraldi used a much wider size range of mud crabs over a longer experimental period [23]. And cannibalism involved mostly small mud crabs, owing to an inverse relationship between molting frequency (i.e., vulnerability) and crab size. The 24-h period for our MPE experiment precluded molting frequency as an influence. Furthermore, density-related interference between mud crabs was likely fostered by using biomass equivalents to represent small-bodied mud crabs [23]. Representing oyster reef predators by many individuals per unit biomass typically shows that predation on oysters is density mediated [14,23]. As such, the exclusion of mud crabs promotes oyster survival in the field [40,48]. Thus, interference and cannibalism among mud crabs would help stabilize the cascade. In contrast, the size-related NCE release effect for mud crabs in our study could weaken the cascade, depending on mud crab size composition. Additionally, oyster consumption was not inhibited by interference among mud crabs in our mixed size TMII experiment. Clearly, size structures of populations of both predators and prey are important in the expression of density-mediated and trait-mediated indirect interactions within oyster-reef food webs.

Through the provision of spatial refuge, habitat complexity can affect the stability of oyster-reef trophic cascades by buffering predation on basal prey [14] and intermediate predators [22], or by mitigating interference [29,36]. Due to its three-dimensional structure containing many crevices, oyster shell offers greater habitat complexity than limestone gravel. Surface area to volume ratios of exposed oyster shell average 3- to 9-fold higher than exposed limestone gravel under field conditions [50]. As a result, consumption of juvenile oysters averaged 1.4-fold higher in limestone gravel than in oyster shell in our study, demonstrating the general stabilizing effect of complex habitat [14]. And equal numbers of oysters in both size classes were consumed across both types of substrate (51% small vs. 49% large in shell; 50% small vs. 50% large in limestone). Presumably, oysters were easier to find in limestone gravel than in oyster shell. In contrast, the stabilizing effect of toadfish within the trophic cascade was weakened somewhat by habitat complexity. In the presence of toadfish, the consumption of oysters by mud crabs was 5-fold higher in oyster shell than in limestone gravel, where mud crabs would have been more vulnerable to predation. Conceivably, the availability of more refuge space for mud crabs facilitated their ability to feed more actively within complex shell substrate. In contrast, Grabowski et al. [14] found that the trophic cascade was stabilized by the availability of refuge space for mud crabs which were less active in the presence of both toadfish and blue crabs together. Likewise, oyster consumption was lower in shell than in limestone when mud crabs were present with both top predators in our study. Other studies have shown that habitat complexity can destabilize the tri-trophic cascade by reducing interference involving intermediate and top predators [29,36]. Thus, the stabilizing influence of habitat complexity on the oyster trophic cascade is context dependent. Effects of habitat complexity are particularly important considering oyster-reef restoration practices, which often entail the use of limestone gravel as substrate [50,51,52,53].

While our MPE experiment roughly simulated oyster reef habitats, it was less realistic than natural conditions in several respects. Selected taxa represented a small subset of the oyster reef community. Consequently, predators were precluded from having mixed diet choices [14]. Predators were restricted to narrow size ranges. Although densities of predators were analogous to the field, they did not vary across treatments. Temperature and salinity were maintained at constant levels and did not fluctuate. Also, the lack of turbid conditions potentially altered perceptual modalities of predators and prey [54]. The presentation of oysters as prey was also unnatural. Predation efficiency likely differs between loose oyster and settled attached oysters due to handling requirements. Survival is lower for cultchless (i.e., lacking settlement substrate) oysters than for attached oysters in the field [40]. Although two size classes of oyster prey were used, the juvenile oyster size distribution was not fully represented. Our experiment also incurred some prey depletion, although full depletion was precluded by the 24-h experimental periods as calibrated by pilot runs. And biological processes acting over periods longer than the 24-h experiments were also precluded, as noted for molting. Survival of oysters and mud crabs may have been lower in our shell substrate consisting of loose whole dead oyster shell than the natural reef matrix. Additionally, restricted movements of experimental animals possibly altered predation efficiency and increased chances of interference within experimental units. Finally, the isolation of experimental units likely enhanced NCEs relative to open field conditions involving currents by concentrating chemical cues [54]. However unrealistic, mesocosm experiments can still provide useful insights into processes operating in the field [54].

The MPE and TMII experiments were incommensurate in ways that provided insights into the expression of NCEs. The magnitude of NCEs varies with the perception of risk in response to many mediating factors [54], including some that differed between the MPE and TMII experiments. The TMII experiment using individual mud crabs was minimized to exclude extraneous influences other than cues emanating from top predators. Notable disparities with the MPE experiment included possible higher concentrations of chemical cues from top predators due to caging stress and lower water volumes, more distinct visual and auditory cues presented by top predators, the lack of confounding by chemical cues from other injured or living mud crabs, the absence of interference from conspecifics or top predators, no confounding due to many cues produced by multiple animals, and greater sensitivity to perceived risk in the absence of substrate. Greater risk sensitivity in the TMII experiment seems likely, as mud crabs were less risk adverse in shell than in limestone in the MPE experiment. Also, mud crab feeding was more stifled in the presence of top predators in the TMII experiment, despite oysters being more readily available than in the MPE experiment. The great disparity between high consumption in the absence of both predators and substrate as opposed to increased inhibition of feeding exacerbated by the lack of substrate may explain a lot of the difference in NCEs between the MPE and TMII experiments. Feeding by intermediate predators was inhibited in the presence of top predators in the MPE and TMII experiments, but feeding was even more inhibited and the relative difference in NCEs was reduced between top predators in the sparse TMII setting. Still, the TMII experiments validated strong NCEs attributable strictly to the inhibition of mud crab feeding induced by top predators in the absence of other mediating factors.

**Conclusions**

Our laboratory experiments revealed several potentially destabilizing effects on the tri-trophic oyster reef cascade that complement previous studies and warrant further testing. First, the intraguild stone crab consumed oysters and mud crabs undeterred by the presence of toadfish. Feeding on oysters by mud crabs was inhibited by the presence of both toadfish and stone crabs, but considerably less so by the latter in the MPE experiment. Consequently, the total effect of all three predator species together weakened the cascade; substantially fewer oysters were consumed when toadfish and mud crabs were the only two predators. While mud crab feeding on oysters was inhibited in the presence of both top predators together, inferred NCEs were stronger for each top predator individually. Weaker inhibition of mud crab feeding was possibly related to higher direct predation on mud crabs by stone crabs than by toadfish. The TMII experiments showed that strong NCEs were attributable strictly to the inhibition of mud crab feeding induced by top predators in the absence of other mediating factors, and that inhibition of mud crab feeding could largely account for NCEs within multiple predator settings,

rather than interference involving other mud crabs or top predators. An inverse relationship between mud crab body size and NCE strength inferred from the TMII experiment potentially poses an additional destabilizing influence on the tri-trophic-cascade. Finally, although habitat complexity generally stabilized the cascade across MPE treatments, greater habitat complexity mitigated the inhibition of mud crab feeding in the presence of toadfish. Still, the toadfish-mud crab-oyster configuration exhibited the lowest oyster consumption, regardless of substrate. Although multiple studies confirm the efficacy of the tri-trophic cascade, some have questioned its relevance under field conditions [40] or have shown that some ecological interactions can weaken it [19, 22, 36, 48]. This study underscores how some ecological interactions can mediate trophic cascades under variable conditions. Our findings must be considered tentative as they emanate from experiments conducted under highly controlled laboratory conditions within a simulated setting. However, our findings provide additional insights about the trophic dynamics of oyster reefs for further testing under more natural conditions.

**Acknowledgements**

This paper is based on research conducted by V.R.S. (Fleer) in fulfillment of the Ph.D. degree in Coastal Sciences from the University of Southern Mississippi, U.S.A. We express our sincere gratitude to M. LaPeyre, R. Leaf, R. Hendon, and K. Dillon for their valuable advice and assistance as Ph.D. committee members. Many thanks are due to the Mississippi Department of Marine Resources for funding the purchase of supplies, and to Scott Rikard and the Auburn Shellfish Laboratory at Dauphin Island for providing oyster spat for this study. Many thanks are due to Chris Lapniewski and Scott McIntosh, for their help with field work. Finally, we thank

two anonymous reviewers for valuable insights that aided the interpretation of this study.

**Author Contributions**

**Conceptualization:** Virginia R. Schweiss, Chet F. Rakocinski.

**Data curation:** Virginia R. Schweiss.

**Formal analysis:** Chet F. Rakocinski.

**Funding acquisition:** Virginia R. Schweiss, Chet F. Rakocinski.

**Investigation:** Virginia R. Schweiss.

**Methodology:** Virginia R. Schweiss, Chet F. Rakocinski.

**Project administration:** Virginia R. Schweiss, Chet F. Rakocinski.

**Resources:** Virginia R. Schweiss, Chet F. Rakocinski.

**Supervision:** Virginia R. Schweiss, Chet F. Rakocinski.

**Validation:** Virginia R. Schweiss.

**Visualization:** Chet F. Rakocinski.

**Writing – original draft:** Virginia R. Schweiss.

**Writing – review & editing:** Chet F. Rakocinski, Virginia R. Schweiss.

**References**

1.	Paine RT. Food web complexity and species diversity. *The American Naturalist*. 1966; 100: 65-75.

2.	Paine RT. Intertidal community structure. Experimental studies on the relationship between a dominant competitor and its principal predator. *Oecologia*. 1974; 15: 93-120.

3.	Schmitt, RJ. Indirect Interactions between prey: Apparent competition, predator aggregation, and habit segregation. *Ecology*. 1987; 68: 1887-1897. doi:10.2307/1939880.

4.	McQueen DJ, France R, Post JR, Stewart TJ, Lean DRS. Bottom-up and top-down impacts on freshwater pelagic community structure. *Ecological Monographs*. 1989; 59: 289–309.

5.	Wootton, JT. 1993. Indirect effects and habitat use in an intertidal community: Interaction chains and interaction modifications. *The American Naturalist*. 1993; 141: 71. doi:10.1086/285461.

6.	Werner EE, Peacor SD. 2003. A Review of trait-mediated indirect interactions in ecological communities. *Ecology*. 2003; 84: 1083–1100. doi:10.1890/0012-9658(2003)084[1083:AROTII]2.0.CO;2.

7.	Grason EW, Miner BG. (2012) Preference alters consumptive effects of predators: Top-down effects of a native crab on a system of native and introduced prey. PLoS ONE. 2012; 7(12): e51322. doi:10.1371/journal.pone.0051322.

8.	Bruno JF, O’Connor MI. Cascading effects of predator diversity and omnivory in a marine food web. *Ecology Letters*. 2005; 8: 1048–56. doi:10.1111/j.1461-0248.2005.00808.x.

9.	O’Connor, NE, Grabowski, JH, Ladwig, LM, Bruno, JF. Simulated predator extinctions: Predator identity affects survival and recruitment of oysters. *Ecology*. 2008; 89: 428–38. doi:10.1890/06-2029.1.

10.	Schmitz, OJ. Direct and indirect effects of predation and predation risk in old‐field interaction webs. *The American Naturalist*. 1998; 151: 327-342.

11.	Carpenter, SR, Kitchell, JF, Hodgson, JR. Cascading trophic interactions and lake productivity. *BioScience*. 1985; 35: 634–39. doi:10.2307/1309989.

12.	Strong, DR. Are trophic cascades all wet? Differentiation and donor-control in speciose ecosystems. *Ecology*. 1992; 73: 747-754. doi:10.2307/1940154.

13.	Schmitz, OJ, Krivan, V, Ovadia, O. Trophic Cascades: The primacy of trait-mediated indirect interactions. *Ecology Letters*. 2004; 7: 153-163.. doi:10.1111/j.1461-0248.2003.00560.x.

14.	Grabowski, JH, Hughes, AR, Kimbro, DL. Habitat complexity influences cascading effects of multiple predators. *Ecology*. 2008; 89: 3413–22. doi:10.1890/07-1057.1.

15.	Sih, A, Bolnick, DI, Luttbeg, B, Orrock, JL, Peacor, SD, Pintor, LM, Preisser, E, Rehage, JS, Vonesh, JR. Predator-prey naïveté, antipredator behavior, and the ecology of predator invasions. *Oikos*. 2010; 119: 610–621.

16.	Hermann, SL, Landis, DA. Scaling up our understanding of non-consumptive effects in insect systems. *Current Opinion in Insect Science*. 2017; 20: 54–60.

17.	Grabowski, JH. Habitat complexity disrupts predator-prey interactions but not the trophic cascade on oyster reefs. *Ecology*. 2004; 85: 995–1004.

18.	Soluk, DA, Collins, NC. Synergistic Interactions between fish and stoneflies: Facilitation and interference among stream predators. *Oikos*. 1988; 52: 94–100. doi:10.2307/3565987.

19.	Fodrie, FJ, Kenworthy, MD, Powers, SP. Unintended facilitation between marine consumers generates enhanced mortality for their shared prey. *Ecology*. 2008; 89: 3268–3274.

20.	Wong, MC, Peterson, CH, Kay, J. Prey size selection and bottom type influence multiple predator effects in a crab-bivalve system. *Marine Ecology Progress Series*. 2010; 409: 143–156. <https://doi.org/10.3354/meps08621>

21.	Crowder, LB, Squires, DD, Rice, JA. Nonadditive effects of terrestrial and aquatic predators on juvenile estuarine fish. *Ecology*. 1997; 78: 1796–1804. [https://doi.org/10.1890/0012-9658(1997)078[1796:NEOTAA]2.0.CO;2](https://doi.org/10.1890/0012-9658(1997)078%5b1796:NEOTAA%5d2.0.CO;2)

22.	Hill, JM, Weissburg, MJ. Habitat complexity and predator size mediate interactions between intraguild blue crab predators and mud crab prey in oyster reefs. *Marine* *Ecology* *Progress Series*. 2013; 488: 209–19. doi:10.3354/meps10386.

23.	Geraldi, NR. Prey size structure diminishes cascading effects by increasing interference competition and predation among prey. *Ecology*. 2015; 96: 2533–2543. <https://doi.org/10.1890/14-1026.1>

24.	Abrams, PA. Implications of dynamically variable traits for identifying, classifying and measuring direct and indirect effects in ecological communities. *The American Naturalist*. 1995; 146: 112–134.

25.	Raffaelli D. Food webs, body size and the curse of the Latin binomial. In: Rooney N, McCann KS, Noakes DLG. (eds) *From Energetics to Ecosystems: The Dynamics and Structure of Ecological Systems.*2007; Springer, Dordrecht.

26.	Freeman, A. Size-dependent trait-mediated indirect interactions among sea urchin herbivores. *Behavioral Ecology*. 2006; 17: 182–187. <https://doi.org/10.1093/beheco/arj014>

27.	Murdoch, WW, Oaten, A. 1975. Predation and population stability. *Advances in Ecological Research*. 1975; 9: 1–131. doi:10.1016/S0065-2504(08)60288-3.

28.	Crowder, LB, Cooper, WE. Habitat stuctural complexity and the interaction between bluegills and their prey (*Lepomis macrochirus*). *Ecology*. 1982; 63: 1802-1813. doi:10.2307/1940122.

29.	Grabowski, JH, Powers, SP. Habitat complexity mitigates trophic transfer on oyster reefs. *Marine Ecology Progress Series*. 2004; 277: 291–95. doi:10.3354/meps277291.

30.	Pace, ML, Cole, JJ, Carpenter, SR, Kitchell, JF. Trophic cascades revealed in diverse ecosystems. *Trends in Ecology and Evolution*. 1999; 14: 483-488. doi:10.1016/S0169-5347(99)01723-1.

31.	Littler, MM, Taylor, PR, Littler, DS. Complex Interactions in the control of coral zonation on a Caribbean reef flat. *Oecologia*. 1989; 80 (3): 331–40. doi:10.1007/BF00379034.

32.	Diehl, S. Fish predation and benthic community structure: The role of omnivory and habitat complexity. *Ecology*. 1992; 73: 1646-1661. doi:10.2307/1940017.

33.	Schriver P, Bøgestrand, J , Jeppesen, E, Søndergaard, M. Impact of submerged macrophytes on fish-zooplankton-phytoplankton interactions. *Freshwater Biology*. 1995; 33: 255-270.

34.	Beukers, JS, Jones, GP. Habitat complexity modifes the impact of piscivores on a coral reef fish population. *Oecologia*. 1997; 114: 50–59. doi:10.1007/s004420050419.

35.	Grabowski, JH, Hughes, AR, Kimbro, DL, Dolan, MA. How habitat setting influences restored oyster reef communities. *Ecology*. 2005; 86: 1926–35. doi:10.1890/04-0690.

36.	Hughes, AR, Grabowski, JH. Habitat context influences predator interference interactions and the strength of resource partitioning. *Oecologia*. 2006; 149: 256–64. doi:10.1007/s00442-006-0439-7.

37.	Swisher, BJ, Soluk, DA, Wahl, DH. Non- additive predation in littoral habitats: influences of habitat complexity. *Oikos*. 1998; 81:30–37.

38.	Griffen, BD, Byers, JE. Partitioning mechanisms of predator interference in different habitats. *Oecologia*. 2006; 146: 608–14. doi:10.1007/s00442-005-0211-4.

39.	Krause, AE, Frank, KA, Mason, DM, Ulanowicz, RE, Taylor, WW. Compartments revealed in food-web structure. *Nature*. 2003; 426: 282–285. <https://doi.org/10.1038/nature02115>

40.	Abbe, GR, Breitburg, DL. The influence of oyster toadfish (*Opsanus tau*) and crabs (*Callinectes sapidus* and Xanthidae) on survival of oyster (*Crassostrea virginica*) spat in Chesapeake Bay: Does spat protection always work? *Aquaculture*. 1992; 107: 21–31. doi:10.1016/0044-8486(92)90047-O.

41.	Shervette, VR, Perry, HM, Rakocinski, CF, Biesiot, PM. Factors influencing refuge occupation by stone crab *Menippe adina* juveniles in Mississippi Sound. *Journal of Crustacean Biology*. 2004; 24: 652–665.

42.	Ives, AR, Cardinale, BJ, Snyder, WE. A synthesis of subdisciplines: predator–prey interactions, and biodiversity and ecosystem functioning. *Ecology Letters*. 2005; 8: 102-116.

43.	Rindone, RR, Eggleston, DB. Predator-prey dynamics between recently established stone crabs (*Menippe* spp.) and oyster prey (*Crassostrea virginica*). *Journal of Experimental Marine Biology and Ecology*. 2011; 407: 216–225. <https://doi.org/10.1016/j.jembe.2011.06.018>

44.	Fleer, VR. *Effects of Trophic Relationships on Oyster Reef Restoration Success in the Mississippi Sound*. 2017; (Doctoral Dissertation) University of Southern Mississippi, Hattiesburg.

45.	Holm, S. A simple sequentially rejective multiple test procedure. *Scandinavian Journal of Statistics*. 1979; 6: 65–70.

46.	Hughes, AR, Rooker, K, Murdock, M, Kimbro, DL. Predator cue and prey density interactively influence indirect effects on basal resources in intertidal oyster reefs. *PLoS ONE*. 2012; 7: e44839. doi:10.1371/journal.pone.0044839.

47.	Macreadie, PI, Geraldi, NR, Peterson, CH. Preference for feeding at habitat edges declines among juvenile blue crabs as oyster reef patchiness increases and predation risk grows. *Marine Ecology Progress Series*. 2012; 466: 145–153. <https://doi.org/10.3354/meps09986>

48.	Johnson, KD, Grabowski, JH, Smee, DL. Omnivory dampens trophic cascades in estuarine communities. *Marine Ecology Progress Series*. 2014; 507: 197–206. <https://doi.org/10.3354/meps10815>

49.	Schneider, FD, Scheu, S, Brose, U. Body mass constraints on feeding rates determine the consequences of predator loss. *Ecology Letters*. 2012; 15: 436–443. <https://doi.org/10.1111/j.1461-0248.2012.01750.x>

50.	KuyKendall, KM, Moreno, P, Powell, EN, Soniat, TM, Colley, S, Mann, R, Munroe, DM. The exposed surface area to volume ratio: Is shell more efficient than limestone in promoting oyster recruitment? *Journal of Shellﬁsh Research*. 2015; 34: 217–225.

51.	Hidu, H, Chapman, S, Soule, PW. Cultchless setting of European oysters *Ostrea*-*edulis* using polished marble. *Proceedings National Shellfisheries Association*. 1974; 65: 13–14.

52.	Soniat, TM, Broadhurst, BC III, Haywood, EL III. Alternatives to clam shell as cultch for oysters and the use of gypsum for the production of cultchless oysters. *Journal of Shellfish Research*. 1991; 10:405–410.

53.	La Peyre, M, Furlong, J, Brown, LA, Piazza, BP, Brown, K. Oyster reef restoration in the northern Gulf of Mexico: Extent, methods and outcomes. *Ocean and Coastal Management.* 2014; 89: 20–28.

54.	Hill, JM, Weissburg, MJ. Predator biomass determines the magnitude of non-consumptive effects (NCEs) in both laboratory and field environments. *Oecologia*. 2013; 172: 79–91.

**Supporting information**

**Figure captions**

**Figure 1 Total number of juvenile oysters consumed across MPE treatments for limestone gravel and oyster shell substrates.** MPE treatments: Control = top predators not present; SC = Stone crab; TF/SC = toadfish and stone crab; MC = mud crab; SC/MC = stone crab and mud crab; TF/MC = toadfish and mud crab; TF/SC/MC = toadfish, stone crab and mud crab.

**Figure 2.** **TMII experiment**. Consumption of Juvenile oysters by three size classes of mud crabs across three caged predator treatments, empty, toadfish and stone crab.

**Data Availability Statement**

Data used for this study were obtained from experiments as described in the Methods and materials section of this article. All data were collected, compiled, curated and validated by V.R. Schweiss. All data reported in this manuscript are publicly available and if this manuscript is accepted for publication, data used for this study, including metadata formats, will be submitted as excel files to the Dryad Digital Repository, at <https://https://datadryad.org/stash/>, for which a Digital Object Identifier (DOI) number will obtained.

**Funding**

Research for this article was conducted as part of a Ph.D. Dissertation by VRS and was not supported by any specific grant from funding agencies in the public, commercial, or not-for-profit sectors. Supplies for this research project were provided to VRS by the Mississippi Department of Marine Resources.

**Competing interests**

The authors have no competing interests.
