# Supplementary material for: Destabilizing effects on a classic tri-trophic oyster-reef cascade
Source: PLoS One. 2020 Dec 15;15(12):e0242965. doi: 10.1371/journal.pone.0242965 (PMC7737904; doi:10.1371/journal.pone.0242965)
Supplement: Attachment — Submitted filename: Response to Reviewers 2.docx [file pone.0242965.s004.docx]

**2^nd^ Revision Responses to Academic Editor – PONE-D-20-05873**

We thank the academic editor for the expert and constructive comments on manuscript PONE-D-20-05873. In response, we made every effort to address the concerns and implement the suggestions. We also revised the manuscript with the objective of reaching PLOS ONE publication standards. Hopefully, we have succeeded. The following details our responses to the concerns and suggestions.

**Comments from the editor:**

**General Comments from the Academic Editor**

*Thank you for submitting your manuscript to PLOS ONE. After careful consideration, we feel that it has merit but does not fully meet PLOS ONE’s publication criteria as it currently stands. Therefore, we invite you to submit a revised version of the manuscript that addresses the points raised during the review process.

In the attached edited version of the revised manuscript, I have added final comments and changes to be made.*

*Please submit your revised manuscript by Sep 26 2020 11:59PM. If you will need more time than this to complete your revisions, please reply to this message or contact the journal office at plosone@plos.org. When you're ready to submit your revision, log on to https://www.editorialmanager.com/pone/ and select the 'Submissions Needing Revision' folder to locate your manuscript file.

Please include the following items when submitting your revised manuscript:*

- *A rebuttal letter that responds to each point raised by the academic editor and reviewer(s). You should upload this letter as a separate file labeled 'Response to Reviewers'.*
- *A marked-up copy of your manuscript that highlights changes made to the original version. You should upload this as a separate file labeled 'Revised Manuscript with Track Changes'.*
- *An unmarked version of your revised paper without tracked changes. You should upload this as a separate file labeled 'Manuscript'.*

*If you would like to make changes to your financial disclosure, please include your updated statement in your cover letter. Guidelines for resubmitting your figure files are available below the reviewer comments at the end of this letter.

If applicable, we recommend that you deposit your laboratory protocols in protocols.io to enhance the reproducibility of your results. Protocols.io assigns your protocol its own identifier (DOI) so that it can be cited independently in the future. For instructions see:* [*http://journals.plos.org/plosone/s/submission-guidelines#loc-laboratory-protocols*](https://nam12.safelinks.protection.outlook.com/?url=http%3A%2F%2Fjournals.plos.org%2Fplosone%2Fs%2Fsubmission-guidelines%23loc-laboratory-protocols&data=02%7C01%7Cchet.rakocinski%40usm.edu%7C37683045451f4a8174f808d83e9f769c%7C7f3da4be2722432ebfa764080d1eb1dc%7C0%7C0%7C637328202182180883&sdata=bAJIJittYlkovNchNONaOuwcMTKCQ4ejMGZTfgqhKw4%3D&reserved=0)

**Responses to specific comments (line numbers with comments correspond to marked up copy with tracks on, line numbers for 2^nd^ revision correspond to unmarked version without tracked changes).**

1) *Line 177. Source reefs do not consume mud crabs. This type of grammatical error is common in the manuscript, and should be corrected.*

Correction acknowledged – document checked for similar errors

Line 157 2^nd^ revision.

2) *Line 193. These are contradictory. Just use complementary.*

Correction accepted

Line 170 2nd revision.

3) *Line 194. Define ‘sparse’.*

’sparse’ defined parenthetically as, “lacking other mediating factors”

Line 171 2nd revision

4) *Line 203. This doesn’t make sense. Why would sand preclude responses?*

Statement rephrased: “The substrate was withheld to limit responses of mud crabs solely to the presence of caged predators.”

Line 179 2nd revision

5) *Line 240. What of the blocking factor in both experiments?*

A statement addressing the blocking factor was was inserted : “Tests of the blocking effect based on separate ANOVAs for each of the two substrate types in which Treatment was a fixed factor and Block was a random factor showed the blocking factor to be unimportant (F=2.116, P = 0.116 oyster shell; F=1.701, P=0.189 limestone gravel).”

Line 215 2nd revision

6) *Line 249. Use r^2^ and two-tailed test.*

Statistical parameters edited accordingly, and n also provided.

Line 220 2nd revision

7) *Line 250. Show data in a table either in text or as a supplement.*

A reference to an S1 Table in the text points to a new table placed in the Supplementary information section which lists mean differences and paired t-tests of small vs. large oysters consumed across treatment levels.

Line 222 2nd revision

8) *Line 260. Do you mean statistically significant?*

Sentence rephrased: “Levene’s and F-tests for unequal error variances and heteroscedasticity were not significant for oyster mortality within the MPE ANOVA.”

Line 227 2nd revision

9) *Line 263. Many units comprise a whole, not the other way around.*

Sentence rephrased: “The ANOVA for the mean number of mud crabs consumed consisted of the four MPE treatments involving mud crabs.”

Line 230 2nd revision

10) *Line 319. These statements are contradictory. Either you do or you don’t have an interaction between substrate and treatment.*

Less contradictory and more nuanced phrasing used: “The mean number of oysters consumed differed for the MPE Treatment and Substrate factors, but not for their interaction (Table 1). Overall, more oysters were consumed in limestone gravel (15.6 ± 1.93 se) than in oyster shell (11.3 ± 1.27 se) across MPE treatments, except for the toadfish/mud crab (TF/MC) treatment for which five-fold more oysters were consumed in the oyster shell than in limestone gravel (t = 3.14; P_1-t_ = 0.007) (Table 2; Figure 1).”

Line 269 2nd revision

P.S. The following justifies the use of post-hoc tests between factor levels of specific interest in the case of nonsignificant interactions, and is excerpted from:

Wei, J, Carroll, RJ, Harden, KK, Wu, G. Comparisons of treatment means when factors do not interact in

two-factorial studies. *Amino Acids*. 2012; 42: 2031–2035. doi:10.1007/s00726-011-0924-0.

“….. based on the general analysis of factor effects, no comparison among the treatment means is usually suggested when the two factors do not interact. However, this does not mean that comparisons among treatment means cannot or should not be made.…..a simple post hoc t test is sufficient to achieve the P value and also provides a greater power in statistical analysis. This new strategy can be used in the future studies involving multiple comparisons of treatment means when there is a nonsignificant interaction between two factors…..Comparison among treatment means when there is no interaction is meaningful for some specific situations.”

11) *Line 358. Table 1. Define this in legend.*

The corrected model term is now defined in the legend: “The corrected model term excludes variability explained by the intercept, thereby constituting an overall test of the dependent variables.”

Line 304 2nd revision

12) *Line 363. Table 2. Add sample sizes so that standard errors can be computed. Better yet, add standard errors and sample sizes.*

Two new columns added to Table 2 to show standard errors and sample sizes.

Line 308

13) *Line 379. Table 3. What is the underline?*

Underlined value replaced by bold value connoting P ≤ 0.05.

Line 324

14) *Line 379. Table 3. Delete the superscripts.*

Done.

Line 336

15) *Line 419.* *This is confusing because Table 3 does not show substrate for all treatments. Please revise.*

This paragraph has been extensively rewritten and expanded as two paragraphs to clarify the interpretation of the interaction effects in the mud crab PERMANOVA, and NCEs relative to mud crabs and oysters as prey.

Line 287

16) *Line 466. Table 4. Define these response variables in the legend.*

The following was appended to the Table 4 legend: “Predator present vs. predator absent values represent numbers of 15 mm oysters consumed during the experiments (out of 10 possible except for the mixed size treatment, which used 20 oysters).”

Line 376

17) *Line 541. Citation?*

Sentence restated and newly cited as [49]: “However, provided toadfish can grow large enough (i.e., > 300 mm TL) [23, 49] to pose a predatory risk to adult stone crabs in the field, crabs of the size used in our experiment may not have entirely outgrown the risk of predation by toadfish. Indeed, the large mouth width of the oyster toadfish equals 20% of its TL [49].

Line 439

18) *Line 562. Define.*

Explicitly defined the first time as “valve length”.

Line 457

19) *Changes by academic editor made throughout the manuscript*

All changes made by the Academic Editor within the revised manuscript were gratefully accepted.
